# In vitro platform to model the function of ionocytes in the human airway epithelium

**DOI:** 10.1186/s12931-024-02800-7

**Published:** 2024-04-25

**Authors:** Marta Vilà-González, Laetitia Pinte, Ricardo Fradique, Erika Causa, Heleen Kool, Mayuree Rodrat, Carola Maria Morell, Maha Al-Thani, Linsey Porter, Wenrui Guo, Ruhina Maeshima, Stephen L. Hart, Frank McCaughan, Alessandra Granata, David N. Sheppard, R. Andres Floto, Emma L. Rawlins, Pietro Cicuta, Ludovic Vallier

**Affiliations:** 1 Wellcome-MRC Cambridge Stem Cell Institute, Jeffrey Cheah Biomedical Centre, University of Cambridge, Puddicombe Way, Cambridge, CB2 0AW UK; 2 Department of Physics, Cavendish Laboratory, University of Cambridge, JJ Thomson Avenue, Cambridge, CB3 0HE UK; 3 Wellcome Trust/CRUK Gurdon Institute, University of Cambridge, Tennis Court Road, Cambridge, CB2 1QN UK; 4 School of Physiology, Pharmacology and Neuroscience, Biomedical Sciences Building, University of Bristol, University Walk, Bristol, BS8 1TD UK; 5 Center of Research and Development for Biomedical Instrumentation, Institute of Molecular Biosciences, Mahidol University, Nakhon Pathom, 73170 Thailand; 6 IRCCS Humanitas Research Hospital, via Manzoni 56, Rozzano, Milan, 20089 Italy; 7 Department of Clinical Neurosciences, Victor Phillip Dahdaleh Heart & Lung Research Institute, University of Cambridge, Papworth Road, Cambridge, CB2 0BB UK; 8 Department of Medicine, Victor Phillip Dahdaleh Heart & Lung Research Institute, University of Cambridge, Papworth Road, Cambridge, CB2 0BB UK; 9 Genetics and Genome Medicine Department, UCL Great Ormond Street Institute of Child Health, London, WC1N 1EH UK; 10 Molecular Immunity Unit, Department of Medicine, University of Cambridge, Cambridge, CB2 0QH UK; 11 Cambridge Centre for Lung Infection, Royal Papworth Hospital NHS Foundation Trust, Cambridge, CB2 0AY UK; 12 Department of Physiology, Development and Neuroscience, University of Cambridge, Tennis Court Road, Cambridge, CB2 1QN UK; 13 BIH Center for Regenerative Therapies, Berlin Institute of Health at Charité, Augustenburger Platz 1, 13353 Berlin, DE Germany; 14 Max Planck Institute for Molecular Genetics, Ihnestraße 63-73, 14195 Berlin, Germany; 15 Cell Therapy and Tissue Engineering Group, Research Institute of Health Sciences (IUNICS), University of Balearic Islands, Palma, 07122 Spain; 16 Health Research Institute of the Balearic Islands (IdISBa), Palma, 07120 Spain

**Keywords:** Airway epithelium, Ionocytes, Human induced pluripotent stem cells, Tissue modelling, FOXI1

## Abstract

**Background:**

Pulmonary ionocytes have been identified in the airway epithelium as a small population of ion transporting cells expressing high levels of *CFTR* (cystic fibrosis transmembrane conductance regulator), the gene mutated in cystic fibrosis. By providing an infinite source of airway epithelial cells (AECs), the use of human induced pluripotent stem cells (hiPSCs) could overcome some challenges of studying ionocytes. However, the production of AEC epithelia containing ionocytes from hiPSCs has proven difficult. Here, we present a platform to produce hiPSC-derived AECs (hiPSC-AECs) including ionocytes and investigate their role in the airway epithelium.

**Methods:**

hiPSCs were differentiated into lung progenitors, which were expanded as 3D organoids and matured by air-liquid interface culture as polarised hiPSC-AEC epithelia. Using CRISPR/Cas9 technology, we generated a hiPSCs knockout (KO) for *FOXI1*, a transcription factor that is essential for ionocyte specification. Differences between *FOXI1* KO hiPSC-AECs and their wild-type (WT) isogenic controls were investigated by assessing gene and protein expression, epithelial composition, cilia coverage and motility, pH and transepithelial barrier properties.

**Results:**

Mature hiPSC-AEC epithelia contained basal cells, secretory cells, ciliated cells with motile cilia, pulmonary neuroendocrine cells (PNECs) and ionocytes. There was no difference between *FOXI1* WT and KO hiPSCs in terms of their capacity to differentiate into airway progenitors. However, *FOXI1* KO led to mature hiPSC-AEC epithelia without ionocytes with reduced capacity to produce ciliated cells.

**Conclusion:**

Our results suggest that ionocytes could have role beyond transepithelial ion transport by regulating epithelial properties and homeostasis in the airway epithelium.

**Supplementary Information:**

The online version contains supplementary material available at 10.1186/s12931-024-02800-7.

## Background

Pulmonary ionocytes were described in 2018 as a small population of airway epithelial cells (AECs) that express high levels of ion channels and transporters, including CFTR (cystic fibrosis transmembrane conductance regulator), the protein mutated in cystic fibrosis (CF) [1–3]. Thus, it has been hypothesised that ionocytes might have a role in the pathogenesis of CF and understanding their function could be key in identifying new therapies for CF and other respiratory diseases. So far, available information on ionocytes and their function in the human airway epithelium is limited. Specific markers for this cell type include FOXI1 (forkhead box I1), high CFTR expression, ASCL3 (achaete-scute family BHLH transcription factor 3) and STAP1 (signal transducing adaptor family member 1) [2]. Ionocytes also express high levels of the vacuolar H^+^ ATP-ase (VATPase), barttin (BSND)/ClC-K channels and the large conductance Ca^2+^-activated K^+^ channel (K_Ca_1.1) [2]. They seem to be more abundant in the nasal epithelium and proximal airways [4, 5] where they are more commonly found in the ducts of submucosal glands [3].

Lineage tracing analysis suggests that ionocytes differentiate from basal cells [2]. By showing that knock out (KO) of *POU2F3* leads to air liquid interface (ALI) cultures with decreased numbers of ionocytes and pulmonary neuroendocrine cells (PNECs), Goldfarbmuren et al. [6] suggested that tuft cells give rise to both ionocytes and PNECs. By contrast, Plasschaert et al. [1] showed that the transcription factor *FOXI1* is sufficient to drive ionocyte differentiation, while the inhibition of Notch signalling in ALI cultures leads to a reduction in their number. This pathway for ionocyte differentiation seems to be conserved between species [7]. More recently, Wang et al. [8] reported no changes in ionocyte marker expression after they overexpressed *NOTCH* in AECs derived from human induced pluripotent stem cell (hiPSCs). This could indicate that lower levels of Notch signalling are needed for ionocyte specification than those required by secretory cells and that signalling is finely tuned to achieve the complex composition of the airway epithelium [9–11]. Finally, Cai et al. [12] demonstrated that the Sonic hedgehog pathway is involved in ionocyte specification by showing that the inhibition of this pathway reduces the amount of ionocytes in culture, while its activation using the chemical agonist SAG (Sonic hedgehog agonist) increases their numbers. Thus, crosstalk between these two signalling pathways seems to be involved in ionocyte specification.

Early studies of the function of ionocytes by Plasschaert et al. showed that reduction of the number of ionocytes could affect CFTR-mediated Cl^−^ currents in Ussing chamber assays [1], which was recently verified by the study of Cai et al. [12]. Additionally, a more recent study demonstrated ionocyte-specific regulation of CFTR by the phosphodiesterase PDE1C [13]. In a *Foxi1* KO mouse model, absence of Foxi1 led to higher mucus viscosity and ciliary beat frequency (CBF), indicating that ionocytes could have a role in regulating airway physiology [2]. This has been further studied in a recent report by Lei et al. [14]. , where they describe a role of ionocytes in fluid and electrolyte absorption, and in a study by Yuan et al. [15] that demonstrates a pivotal role for ionocytes in homeostatic mechanisms regulating airway surface liquid (ASL) volume, pH and viscosity and mucociliary clearance. Furthermore, the observation that ionocytes have cellular extensions [2, 16], suggests the hypothesis that they could interact directly with other AEC types. However, the precise mechanisms by which ionocytes control these multiple functions are still not fully understood.

The challenge to further understand ionocyte function in human lungs is aggravated by the lack of appropriate models and the limited availability of primary tissue. AECs derived from hiPSCs (hiPSC-AECs) could provide unique opportunities for respiratory research since hiPSCs can grow indefinitely while maintaining their capacity to differentiate into any cell type. However, the differentiation of hiPSCs into AECs lacks standardised protocols and different methods often lead to divergent results [17–19]. Until recently, protocols failed to consistently produce rare AECs such as ionocytes [18]. Hor et al. published a protocol to generate PNECs from hiPSCs in vitro, without identifying ionocytes in their cultures [20]. In a recent report, Wang et al. [8] identified ionocytes in their hiPSC-AEC cultures using a protocol with 3 sorting steps which extended the length of the protocol to almost 80 days. Here, we present a platform to study the role of ionocytes in the airway epithelium in vitro using hiPSCs. We describe a protocol which produces AECs including ionocytes within 60 days and then perform loss of function experiments. Our results show that the KO of FOXI1 in hiPSCs using CRISPR/Cas9 reduced the number of ciliated cells after hiPSC-AEC maturation, indicating that ionocytes could be important in lung lineage specification and homeostasis.

## Methods

Full descriptions of the methods used to differentiate hiPSCs into AECs and evaluate them biochemically and functionally are provided in the Supplementary Materials and Methods.

### hiPSC differentiation to AECs

To derive AECs from hiPSCs, we used FS13B hiPSC lines generated as described previously [21] and the CF17/NKX2.1-GFP hiPSC line (kindly gifted by UTHEALTH and Dr. Jed Mahoney, Cystic Fibrosis Foundation lab, Lexington, MA, USA). hiPSCs were differentiated by driving cells through definitive endoderm and anterior foregut endoderm to reach a lung progenitor state. At day 16 of differentiation, cells were sorted to enrich for NKX2.1 expressing progenitors using anti-carboxypeptidase-M (CPM) antibody [17], anti-CD26/anti-CD47 sorting strategy [22] or sorting for GFP: NKX2.1 reporter cells [18]. Sorted cells were seeded in 3D Matrigel domes for expansion and cryopreservation. After at least 8 days of growth under expansion conditions, cells were seeded on Transwell® inserts to form mature polarised airway epithelia. Once cells were confluent in the Transwell®, medium bathing the apical membrane was removed to form an ALI. After 28 days of ALI culture, hiPSC-AEC epithelia were characterised biochemically and functionally.

### Analysis of mRNA and protein expression

Reverse transcription-quantitative polymerase chain reaction (RT-qPCR), immunofluorescence staining and Western blotting were performed to characterise mRNA and protein expression at different stages of the protocol and to investigate the effects of *FOXI1* KO.

### Lung progenitor transplantation into a mouse model of airway injury

Experiments using a mouse model of airway injury were approved by local ethical review committees and conducted according to Home Office project license PPL PEEE9B8E4 (Emma L. Rawlins, University of Cambridge). For these experiments, 9 male 9-week-old immune-compromised *NOD-scid-IL2rg*^*−/−*^ (NSG; RRID: IMSR_JAX:005557) mice were used [23, 24]. Mice were treated with 2% polidocanol oropharyngeally and transplanted with a suspension of 1 million GFP + hiPSC-derived lung progenitor cells on the next day. At different time points (1, 7 or 10 days) after cell transplantation, mice were sacrificed and tracheas harvested for wholemount immunofluorescence staining to visualise cells.

### **CRISPR/Cas9-based*FOXI1*KO and phenotypical assays**

Single guide RNA (sgRNA) and CRISPR/Cas9 were used to KO *FOXI1* in hiPSCs. The functional consequences of *FOXI1* KO were evaluated using hiPSC-AEC epithelia and pH and transepithelial resistance (R_t_) measurements, high-speed microscopy analysis of ciliary dynamics and Ussing chamber studies of epithelial ion transport.

### Statistical analysis

Results are expressed as means ± SD of *n* observations. Statistical analyses were performed either using Prism 9 (GraphPad Software Inc., San Diego, CA, USA) or SigmaPlot 14 (Systat Software Inc., San Jose, CA, USA). The type of statistical analysis performed in each experiment and the number of replicates used are described in the figure legends. Differences were considered statistically significant when *P* < 0.05. Significance in each analysis is represented by * *P* < 0.05, ** *P* < 0.01, *** *P* < 0.001, **** *P* < 0.0001, ns = not significant.

## Results

### Method to differentiate hiPSCs into AECs including ionocytes

**Fig. 1:**
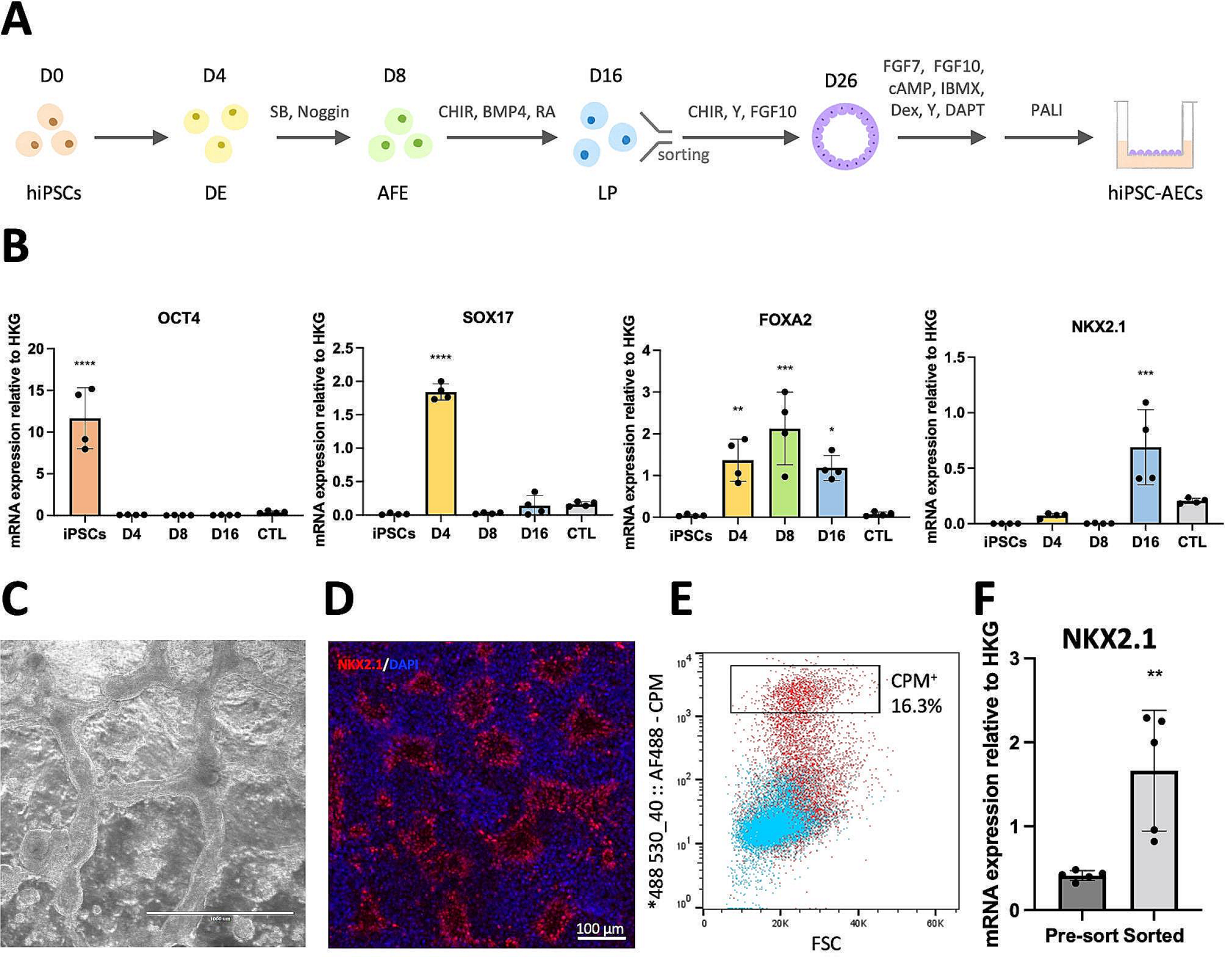
hiPSCs differentiate into lung progenitors in 16 days. **A**: Diagram of the differentiation protocol, fluorescence activated cell sorting, expansion in 3D organoids and maturation at an ALI to form hiPSC-AECs. Abbreviations: AFE, anterior foregut endoderm; BMP4, bone morphogenetic protein 4; CHIR, CHIR-99021; DAPT, (2 S)-N-[2(3,5-Difluorophenyl)acetyl]-L-alanyl-2-phenyl-glycine 1,1-Dimethylethyl ester; DE, definitive endoderm; FGF7/10, fibroblast growth factor 7/10; IBMX, 3-isobutyl-1-methylxanthine; LP, lung progenitor; PALI, PneumaCult™-ALI Medium; RA, retinoic acid; SB, SB431542; Y, Y-27632. **B**: Relative mRNA expression of key markers at different time points during differentiation. The control (CTL) is human trachea total mRNA. Filled circles represent individual values and columns are means ± SD (*n* = 4 independent experiments). *P < 0.05, **P < 0.01, ***P < 0.001, ****P < 0.0001 vs. D0; one-way ANOVA with Tukey’s post-test. **C**: Brightfield image of cells after 16 days of differentiation. The scale bar is 1000 μm. **D**: Immunofluorescence staining showing lung progenitor marker NKX2.1 expression (red) and nuclear marker DAPI (blue) on day 16 of differentiation. The scale bar is 100 μm. **E**: Flow cytometry panel showing levels of expression of CPM at day 16 of differentiation (red). The population labelled CPM + was sorted for enrichment of NKX2.1-expressing lung progenitors. HiPSCs stained with anti-CPM antibody served as a negative control (blue). **F**: Enrichment of NKX2.1 mRNA expression after sorting of CPM + cells. Filled circles represent individual values and columns are means ± SD (*n* = 5 independent experiments). **P < 0.01 vs. pre-sorted; Student’s t-test

**Fig. 2:**
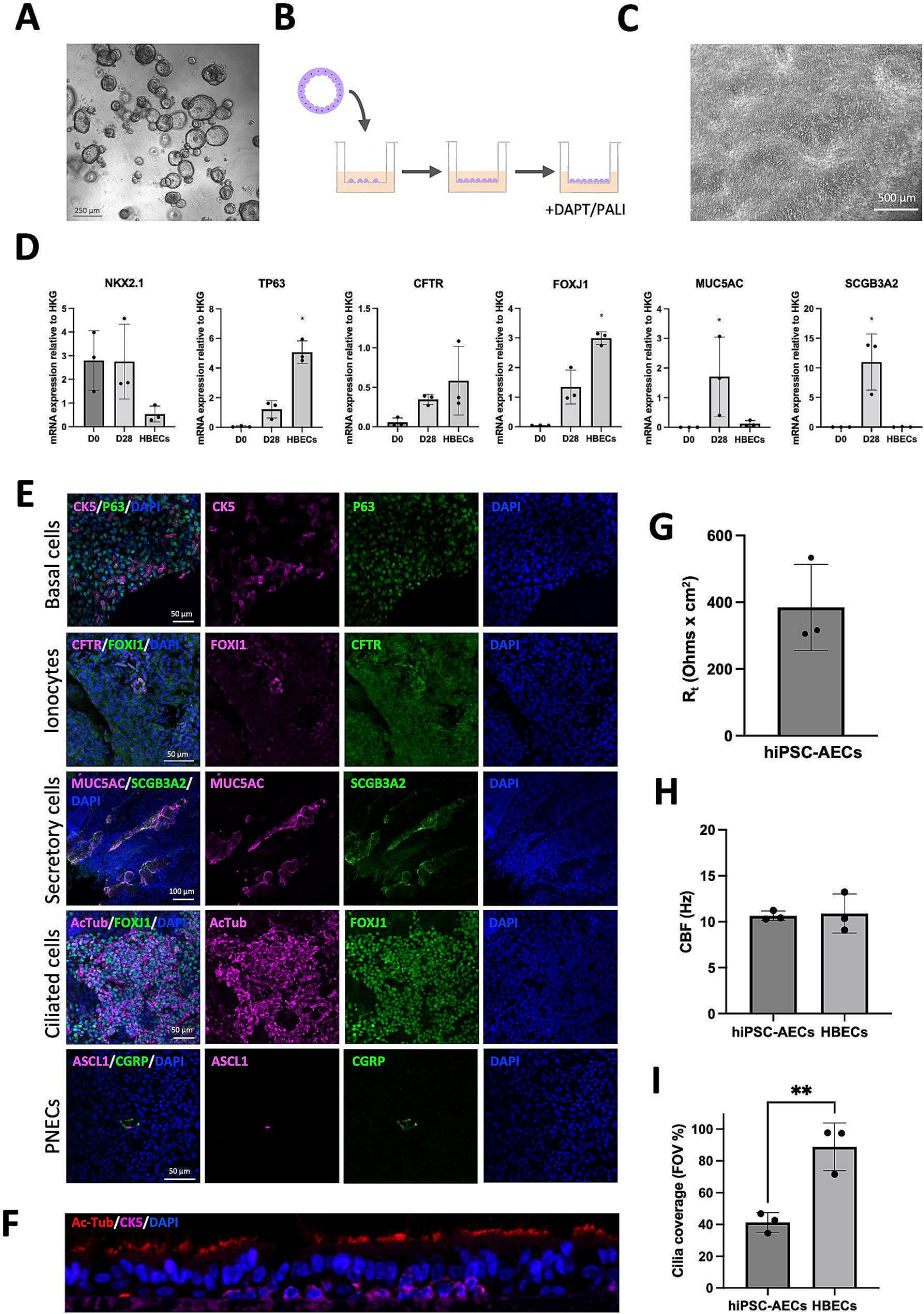
ALI culture induces differentiation towards mature AECs with similar properties to HBECs. **A**: Brightfield image of lung progenitors in 3D organoid culture. The scale bar is 250 μm. **B**: Schematic of ALI culture. Organoids were dissociated and cells seeded in Transwell® inserts and cultured with medium on both sides. Once cells were confluent, medium from the top compartment was removed to form an ALI with the apical membrane of cells in contact with air. DAPT was added to the maturation medium in the bottom compartment and the cells cultured for a further 14 days, followed by another 14 days of culture with PALI medium. **C**: Brightfield image of hiPSC-AECs in a Transwell® insert after establishing an ALI. The scale bar is 500 μm. **D**: mRNA expression of AEC markers in cells in expansion conditions (D0) and in ALI culture (D28). ALI cultured HBECs were used as a control. Filled circles represent individual values and columns are means ± SD (*n* = 3 independent experiments); *P < 0.05 vs. D0; one-way ANOVA with Tukey’s post-test. **E**: Immunocytochemical analysis of mature AEC markers in ALI cultures. The scale bars are 50–100 μm as indicated. **F**: Immunofluorescence staining of a histological section through an ALI culture showing polarization of the airway epithelium. Cilia at the apical side are labelled with Acetylated tubulin (AcTub) and mature basal cells on the basal side are labelled with CK5. **G**: R_t_ measurements of polarized epithelia formed by hiPSC-AECs. Filled circles represent the average of three readings of the same sample and columns are means ± SD (*n* = 3 independent experiments). **H**: Ciliary beat frequency (CBF) measurements in hiPSC-AECs and HBECs. Filled circles represent the average of 20 FOVs from one sample and columns are means ± SD (*n* = 3 independent experiments); Student’s t-test. **I**: Area covered by cilia in hiPSC-AECs ALI cultures is significantly smaller compared to HBECs. Filled circles represent the average of 20 FOVs from one sample and columns are means ± SD (*n* = 3 independent experiments); **P < 0.01; Student’s t-test

For this study, we used two different hiPSC lines or genetic backgrounds: the previously described FS13B hiPSCs [21] and the CF17/NKX2.1-GFP, which has a GFP reporter for NKX2.1. We first differentiated these two hiPSCs lines into AECs following a natural path of development including definitive endoderm, anterior foregut endoderm and lung progenitors (Fig. 1A). RT-qPCR analyses confirmed the mRNA expression of specific markers for each stage (Fig. 1B and S1A) and cells formed a characteristic network pattern after 16 days of differentiation (Fig. 1C and D). The resulting lung progenitors were then sorted using anti-CPM staining to enrich for NKX2.1 expressing cells in FS13B cells (Fig. 1E and F) while CF17/NKX2.1-GFP cells were sorted for NKX2.1-GFP expression. To expand lung progenitors, sorted cells were grown as 3D organoids (Fig. 2A) in medium supplemented with the GSK3b inhibitor CHIR-99021, the Rho-associated protein kinase inhibitor Y-27632 and fibroblast growth factor 10 (FGF10). These organoids could be cryopreserved and thawed for further experiments while maintaining the expression of lung progenitor markers (Figure S1B). In some instances, organoids were maintained for up to 8 passages or + 6 passages after thawing without losing NKX2.1 expression (Figure S1C). Overall, our approach allowed the production and the expansion of lung progenitors in vitro thereby bypassing the need to systematically differentiate hiPSCs.

AEC maturation was performed by dissociating the organoids and seeding lung progenitors in Transwell® inserts. Once confluent, ALI was established and cells were differentiated for an additional 28 days (Fig. 2B and C). To promote the differentiation of ciliated cells, the Notch pathway inhibitor (2 S)-N-[2(3,5-Difluorophenyl)acetyl]-L-alanyl-2-phenyl-glycine 1,1-Dimethylethyl ester (DAPT) was added to the Maturation Medium for the first 14 days after initiating ALI culture. From day 14, PneumaCult™-ALI (PALI) Medium was used to further promote ciliation. The resulting epithelia showed an increase in the expression of *TP63*, *CFTR*, *FOXJ1, MUC5AC* and *SCGB3A2* (Fig. 2D) and maintained expression of epithelial markers (Figure S1D). The presence of basal cells (CK5, p63), secretory cells (MUC5AC, SCGB3A2), ciliated cells (FOXJ1, acetylated tubulin (AcTub)), PNECs (ASCL1, CRP) and ionocytes (FOXI1, CFTR high expression, BSND) was confirmed by immunostaining (Fig. 2E and S1E). Importantly, the epithelium was polarised, with cilia (AcTub) located on the apical side and basal cells (CK5) at the basal side of the epithelium (Fig. 2F). Finally, functional analyses confirmed that the hiPSC-AEC epithelium had R_t_ values comparable to that of primary AECs [25] (Fig. 2G). Analysis of CBF by a robust Fourier Transform method [26], described in the Supplementary Materials and Methods, showed that the cilia in hiPSC-AEC cultures beat at a frequency comparable to that of primary human bronchial epithelial cells (HBECs) (Fig. 2H). The same analysis indicated that hiPSC-AECs were covered by fewer cilia than HBEC cultures (Fig. 2I), consistent with RT-qPCR results for *FOXJ1* expression (Fig. 2D). Taken together, these results show that our protocol allows the production of a polarised airway epithelium containing a diversity of cell types, including ionocytes.

### The engraftment capacity of hiPSC-derived lung progenitors

**Fig. 3:**
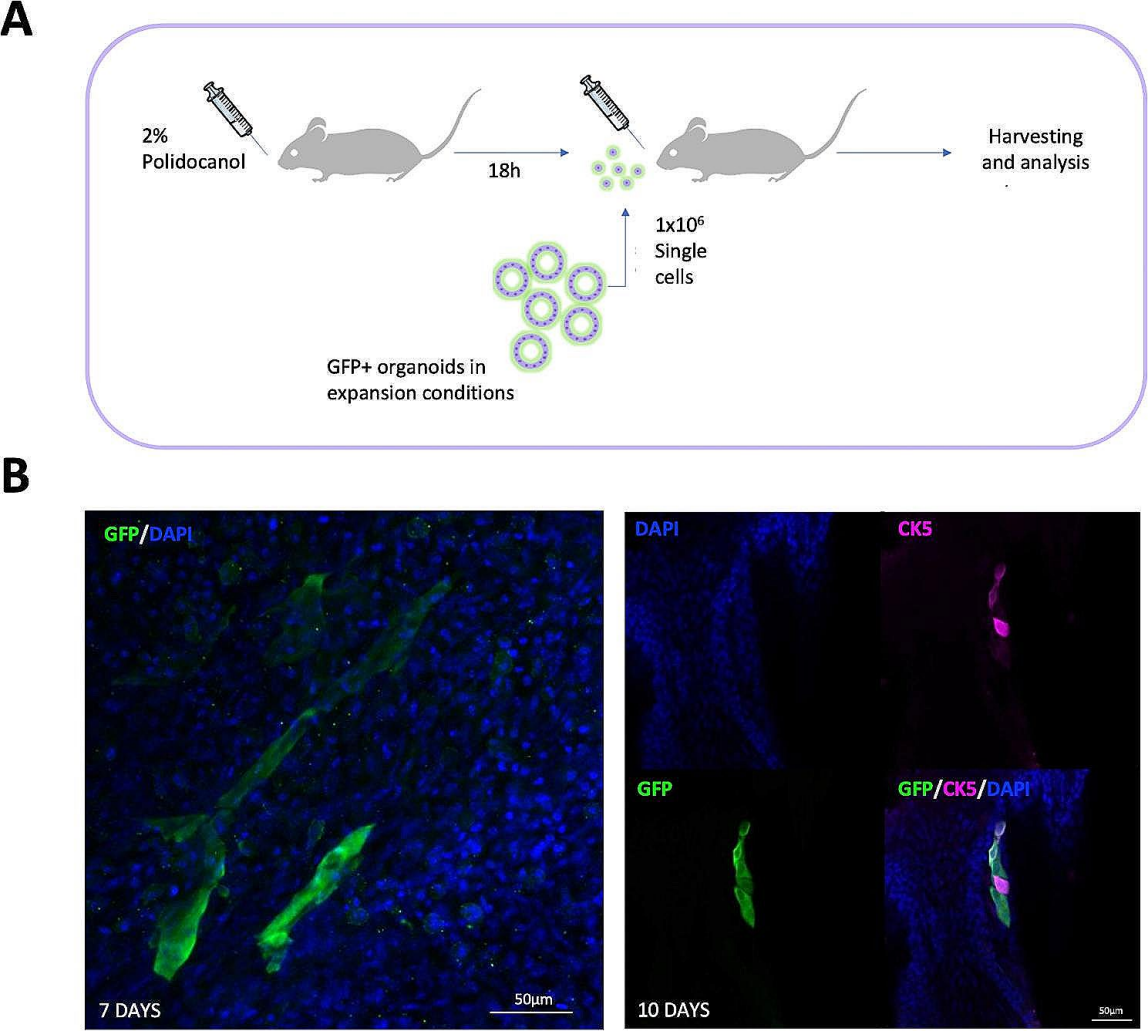
hiPSC-derived lung progenitors engraft in an in vivo mouse model of airway injury. **A**: Schematic of the cell transplantation procedure. Mice were anesthetized and 30 µl of 2% Polydocanol was administered oropharyngeally. After 18 h, mice were anesthetised again and 30 µl of sterile PBS with 1% BSA and 1 million GFP + hiPSC-derived lung progenitors were administered to the back of the throat. Tracheas were harvested at different time points for analysis. **B**: Representative wholemount immunofluorescence staining showing GFP and DAPI at 7 days after transplantation (left) and GFP, human CK5 and DAPI at 10 days after transplantation (right). The scale bars are 50 μm

hiPSC-derived lung progenitors have been previously successfully transplanted into the respiratory airways of murine models [27–29], highlighting their potential in regenerative medicine. To further demonstrate the functionality of our cells, we explored the engraftment potential of our hiPSC-derived airway progenitors in vivo with a short-term transplantation experiment. We generated lung progenitors from GFP-expressing hiPSC lines (FS13B GFP) and GFP + CPM + sorted cells were cultured as 3D organoids for 8 days before cryopreservation. After thawing and expansion for at least 8 additional days, GFP + organoids expressed similar levels of the lung progenitor and basal cell markers *NKX2.1* and *TP63* when compared to passage 0 organoids (Figure S2A). These organoids were then dissociated into a single cell suspension and 1 million cells were transplanted oropharyngeally into the tracheas of mice that had been topically treated with polidocanol 18 h before transplantation (Fig. 3A). Tracheas were harvested at 1, 7 or 10 days after transplantation and we observed GFP + cells in the tracheas of the 9 mice that had received hiPSC-derived cells (Fig. 3B and Figure S2B). The appearance of clusters of cells indicates that the cells replicated after engraftment (Fig. 3B left). Interestingly, GFP + cells co-expressed CK5 at 7 and 10 days after transplantation (Fig. 3B right), indicating that lung progenitors generated with our approach can not only survive in a mouse model of acute airway injury, but also differentiate towards basal cells. Although longer time points would be needed to assess the full regeneration and differentiation potential of these cells, these results confirm their engraftment capacity.

### **KO of*FOXI1*in hiPSCs leads to hiPSC-AECs lacking ionocytes**

**Fig. 4:**
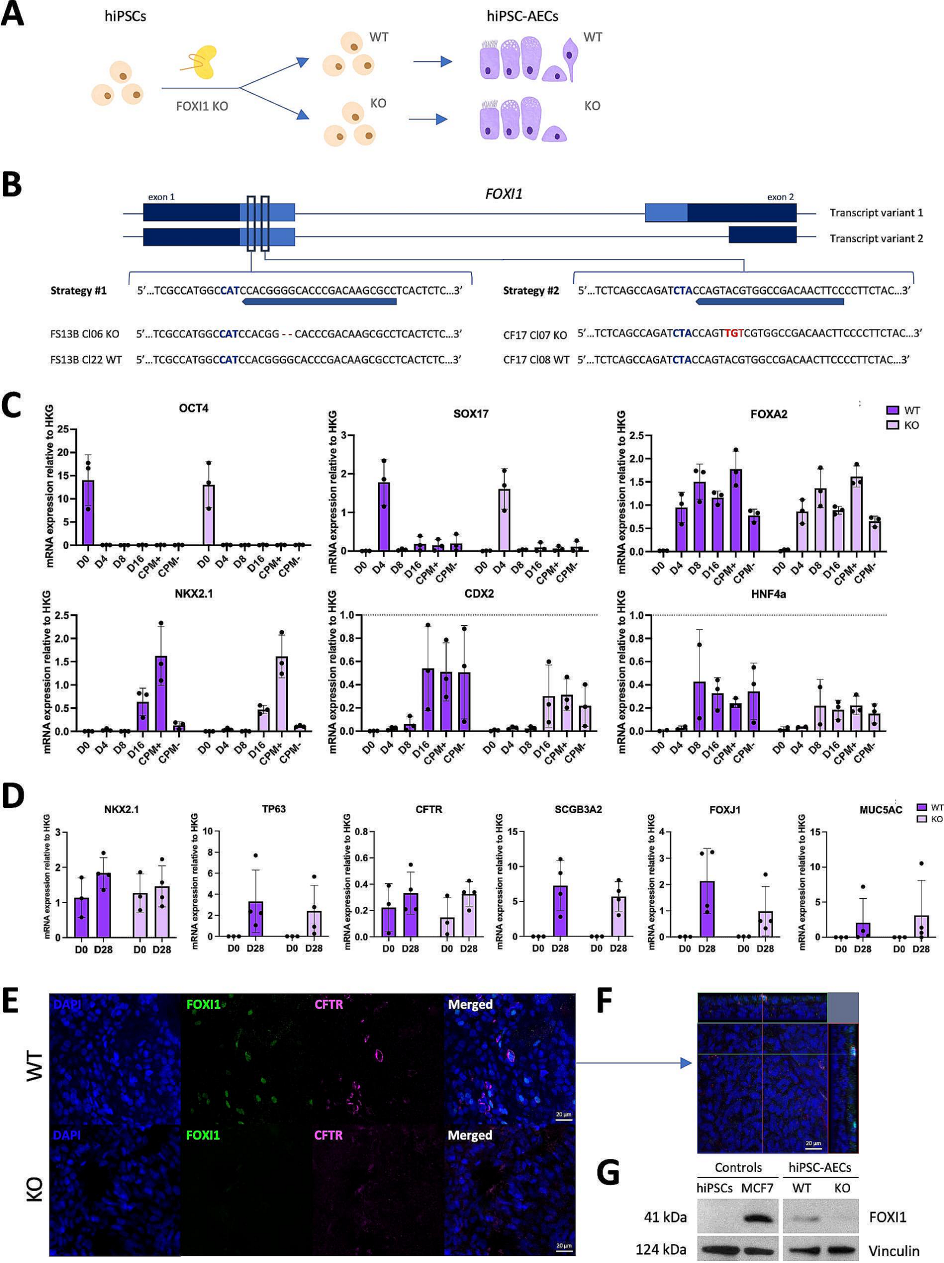
*FOXI1* knock-out (KO) in hiPSCs leads to hiPSC-AEC cultures lacking ionocytes. **A**: *FOXI1* gene targeting and differentiation strategy. Following *FOXI1* KO in hiPSCs, WT and KO clones were selected from the targeted pool and differentiated in parallel towards AECs. *FOXI1* KO cells were not expected to generate ionocytes. Targeted but unedited WT cells served as an isogenic control. **B**: Each hiPSC line was targeted with a different sgRNA (Strategy #1 for FS13B and Strategy #2 for CF17/NKX2.1-GFP), testing two different genetic backgrounds and two different targeting strategies in the same study. The diagram shows the sgRNA used on each line to target the DNA binding domain in Exon 1, the PAM region highlighted in blue and the indels in the selected KO clones in red. **C**: Relative mRNA expression of key markers at different time points during the first stages of differentiation. Filled circles represent individual data points and bars are means ± SD (*n* = 3 independent experiments); two-way ANOVA with Sidak multiple comparison test. The dotted line indicates the level of the normalized reference-gene expression average value. **D**: Relative mRNA expression of key mature AEC markers of cells in expansion (D0) and after maturation in ALI cultures (D28). Filled circles represent individual data points and bars are means ± SD (*n* = 3 independent experiments), two-way ANOVA with Sidak multiple comparison test. **E**: Representative immunocytochemical staining of FOXI1, CFTR and DAPI in mature hiPSC-AECs after 28 days in ALI culture in *FOXI1* WT and KO cells. The scale bar is 20 μm. **F**: Z-stack panel with orthogonal views of *FOXI1* WT cells from E. **G**: Cropped representative Western blot images of FOXI1 expression in WT and KO hiPSC-AECs (right panel), undifferentiated hiPSCs were used as a negative control and MCF7 cells were used as a positive control (left panel). Vinculin was used as a loading control

Based on the results generated above, we decided to use our platform to study the importance of ionocytes during the formation of the human airway epithelium. Of note, genetic studies in the mouse have shown that FOXI1 is necessary for the generation of ionocytes in vivo [2]. Thus, we hypothesised that the absence of FOXI1 will stop the production of ionocytes in vitro (Fig. 4A). Using CRISPR/Cas9 genome editing, we generated two hiPSC KOs for the *FOXI1* gene by designing sgRNAs that target the DNA binding domain of *FOXI1*, which is found in exon 1 and is shared by both transcript variants of the gene (Fig. 4B). This approach generated hiPSC lines carrying a loss of function mutation caused by an early stop codon (Figure S3A) as confirmed by genotyping using PCR and Sanger sequencing. Importantly, each of the hiPSC lines was targeted with a different sgRNA to rule out off-target effects, while unedited clones that had gone through the same targeting and clone isolation process were used as isogenic controls (Fig. 4B). The resulting *FOXI1*^−/−^ hiPSCs were then differentiated in parallel with their isogenic wild-type (WT) counterparts. There were no statistically significant differences in the expression of specific markers at key timepoints between the WT and KO up to day 16 of differentiation (Fig. 4C). Thus, the absence of FOXI1 does not affect lung progenitor production. Lung progenitor cells were then enriched by sorting (Fig. 4C) and the resulting organoids were further differentiated using ALI cultures after expansion. After 28 days of culture, WT and KO epithelia showed similar levels of airway markers including *NKX2.1*, *TP63*, *CFTR*, *SCGB3A2*, *FOXJ1* and *MUC5AC* (Fig. 4D). However, the absence of *FOXI1* seemed to induce a limited decrease in the expression of *FOXJ1* in ALI cultures (Fig. 4D and S3B). Of note, we could not detect changes in *FOXI1* expression by RT-qPCR as ionocytes represent only 0.5–1.5% of the epithelium. Immunofluorescence analyses showed the absence of ionocytes in the *FOXI1* KO ALI cultures in contrast to the presence of FOXI1 + CFTR high-expressing ionocytes in WT ALI cultures (Fig. 4E and F). Furthermore, Western blotting indicated absence of FOXI1 protein in *FOXI1* KO ALI cultures compared to its presence in WT epithelia (Fig. 4G and S3C). Together, these data show that *FOXI1* KO leads to hiPSC-AEC epithelia without ionocytes and that their absence does not affect the early differentiation of the lung epithelium.

### **KO of*FOXI1*reduces ciliation of hiPSC-AECs**

**Fig. 5:**
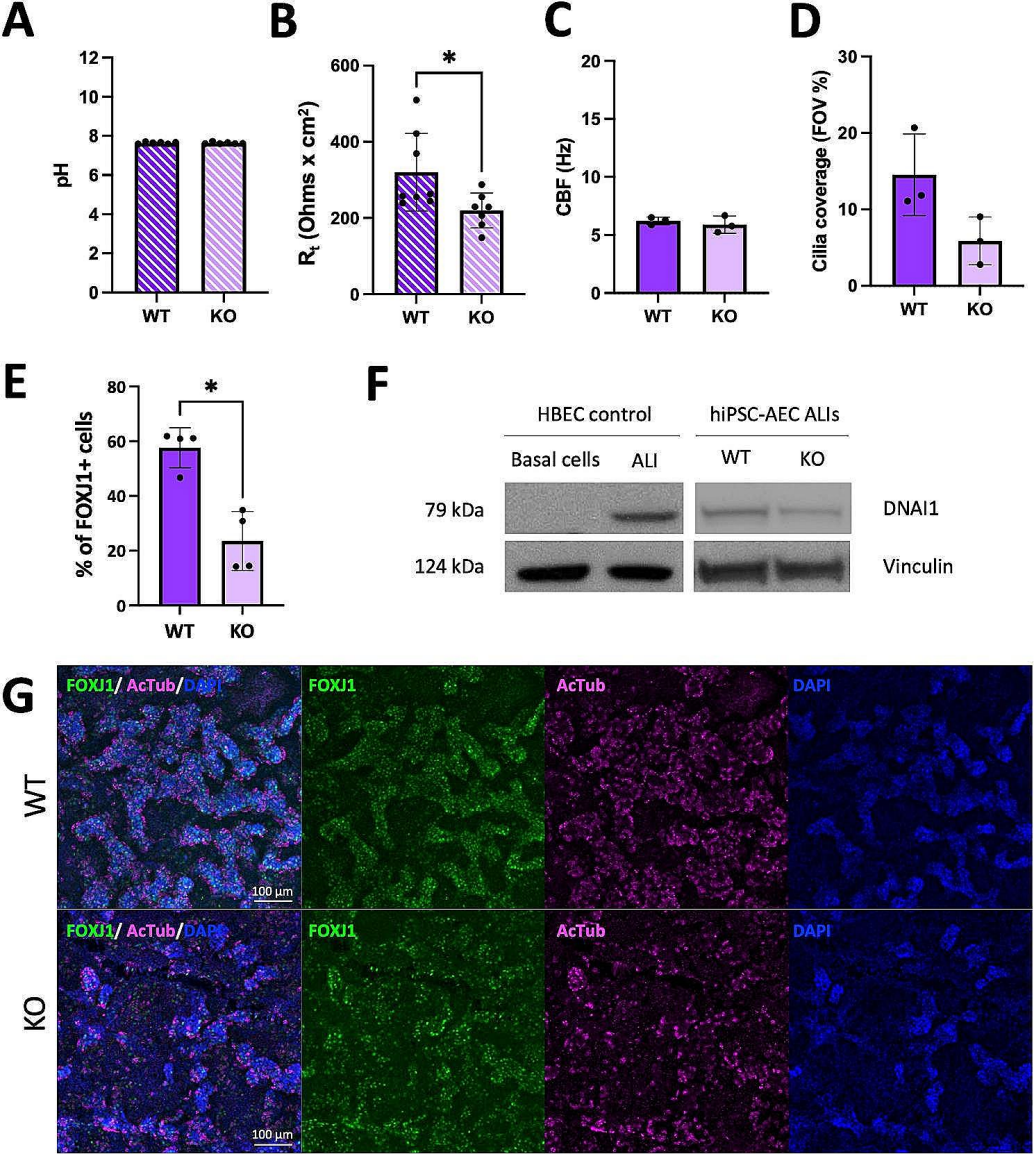
Functional assays reveal that *FOXI1* KO leads to decreased numbers of ciliated cells in hiPSC-AECs. **A**: Airway surface liquid (ASL) pH of mature *FOXI1* WT and KO hiPSC-AECs. Filled circles represent individual values and bars are means ± SD (*n* = 6 consists of 3 independent experiments, 2 biological replicates per experiment); Mann-Whitney test. **B**: Transepithelial resistance (R_t_) of mature *FOXI1* WT and KO hiPSC-AEC ALI cultures. Filled circles represent the average of 3 technical replicates (measurements) and bars are means ± SD (*n* = 6 ALIs from 3 independent experiments, 2 biological replicates per experiment). * *P* < 0.05; Mann-Whitney test. **C**: Ciliary beat frequency (CBF) of *FOXI1* WT and KO hiPSC-AEC ALI cultures. Filled circles represent the average of values obtained from 5–20 FOVs with > 5% of coverage from one sample and bars are means ± SD (*n* = 3 independent experiments); Student’s t-test. **D**: Area covered with motile cilia in *FOXI1* WT and KO hiPSC-AEC ALI cultures. Filled circles represent the average of up to 20 FOVs from one sample and bars are means ± SD (*n* = 3 independent experiments); Student’s t-test. **E**: Flow cytometry analysis of the amount of FOXJ1 + ciliated cells in *FOXI1* WT and KO hiPSC-AEC ALI cultures. Gating was performed compared to stained hiPSC controls. Filled circles represent individual values and bars are means ± SD (*n* = 4 independent experiments); *P < 0.05; Mann-Whitney test. **F**: Cropped representative Western blot images show the expression of the ciliated cell marker DNAI1 in mature *FOXI1* WT and KO hiPSC-AECs. Primary basal cells and HBEC ALI cultures served as negative and positive controls, respectively. Vinculin served as a loading control. **G**: Representative immunofluorescence staining of FOXJ1, acetylated tubulin (AcTub) and DAPI in *FOXI1* WT and KO hiPSC-AECs. The scale bar is 100 μm

We next tested whether the KO of *FOXI1* could affect the function of hiPSC-AECs beyond that of CFTR function, which has already been extensively studied [1, 2, 12–14]. In this study, we first assessed the effect of *FOXI1* KO on the pH of the ASL in hiPSC-AEC ALI cultures and we confirmed that there were no statistical differences between *FOXI*1 WT and KO cultures (Fig. 5A). We next evaluated whether the KO of *FOXI1* impaired the barrier properties in hiPSC-AEC ALI cultures by measuring R_t_. Although *FOXI1* KO epithelia had significantly reduced R_t_ values compared to those of *FOXI1* WT epithelia (Fig. 5B), Ussing chamber studies revealed that they functionally expressed the epithelial Na^+^ channel (ENaC), the Ca^2+^-activated Cl^−^ channel TMEM16A and CFTR (Figure S6). Next, we assessed cilia coverage and motility as described in Supplementary Materials and Methods. *FOXI1* KO ALI cultures showed a similar CBF to their WT counterparts (Fig. 5C and S4). Intriguingly, the coverage of cilia in *FOXI1* KO epithelia was reduced (Fig. 5D and S4). Although this difference was not statistically significant, it suggested that either the number or function of ciliated cells was decreased in *FOXI1* KO ALI cultures. To distinguish between these possibilities, we performed flow cytometry analyses and observed that the absence of ionocytes resulted in a significant reduction in the number of FOXJ1 expressing cells (Fig. 5E and S5A). The reduced number of ciliated cells in *FOXI1* KO cells was validated with hiPSC-AECs from two other *FOXI1* KO hiPSC clones of the same genetic background (Figure S5B and S5C) and with the clones from the second genetic background (Figure S5D and S5E). Because the FOXJ1 + cell number decrease was not as striking in the second genetic background (Figure S5D and S5E), we further investigated this phenotype in CF17/NKX2.1-GFP cells by assessing the expression of mature ciliated cell markers both at protein and mRNA levels. Western blot analysis indicated a decrease of DNAI1 in *FOXI1* KO cells compared to their WT controls (Fig. 5F). RT-qPCR analysis showed decreased expression of the ciliated cell markers *NEK10, DNAH5* and *CP110*, but only the differences in *DNAH5* and *CP110* expression were statistically significant (Figure S5F). Finally, immunofluorescence staining confirmed the presence of FOXJ1 + AcTub + ciliated cells in *FOXI1* KO ALI cultures, but these had a more scattered distribution compared to their WT controls (Fig. 5G). Taken together, these results suggest that ionocytes and/or the expression of *FOXI1* could be involved in the production of functional ciliated cells and could be necessary to establish the normal cellular composition of the lung epithelium.

## Discussion

In this study, we described a protocol to differentiate hiPSCs not only into the most abundant cell types of the airway epithelium (basal, ciliated and secretory cells) but also into PNECs and ionocytes. To our knowledge, this is the first report of hiPSC-AECs including these rare cell types in the same culture system.

To date, only one other protocol for hiPSC-AEC differentiation producing ionocytes has been published [8]. In that study, Wang et al. reported the presence of FOXI1 + ionocytes using a protocol that requires 3 subsequent sorting steps. Thus, the generation of hiPSC-AEC cultures with ionocytes has proven challenging. One of the reasons for this might be the use of specialised media developed to produce highly ciliated cultures, which probably contain inhibitors of Notch that could reduce the presence of ionocytes [1]. By contrast, our protocol is based on a chemically defined medium combined with short-term culture with PneumaCult™-ALI Medium. This combination leads to hiPSC-AEC cultures that might be less ciliated, but that contain ionocytes expressing FOXI1 and high levels of CFTR or co-expressing FOXI1 and BSND.

We used our hiPSC-AEC cultures to study the impact of *FOXI1* KO on the development and functionality of the airway epithelium. Although ionocytes constitute a rare population in the epithelium, several studies have shown that their impairment can lead to significant phenotypes [1, 2, 12–14]. Consistent with previous results [2], we found that the KO of *FOXI1* does not impact the pH of ASL in ALI cultures. By contrast, we found that *FOXI1* KO impacts R_t_ values of hiPSC-AEC epithelia. The lower R_t_ values of *FOXI1* KO epithelia might be due to impaired epithelial barrier function and/or increased numbers/activity of ion channels. According to Pou Casellas et al. [30], transcriptional analysis of ionocytes revealed their involvement in various signalling pathways, including those involving occludin and junctional adhesion molecules, which could potentially explain why their absence affects the formation of a tight epithelial barrier. Additionally, Yuan et al. [15] reported the compensatory overexpression of ion and water channel encoding genes in airway cultures from a *FOXI1* KO ferret model. Although our results differ from those published by Goldfarbmuren et al. [6] and Lei et al. [14], who reported increased R_t_ in *FOXI1* KO cultures, their data are based on mosaic KOs, while Yuan et al. [15] did not report R_t_ measurements. The different R_t_ values of *FOXI1* KO airway epithelia are reminiscent of earlier reports about the effects of the predominant CF-causing *CFTR* variant F508del on R_t_. LeSimple et al. [31] found that epithelia heterologously expressing F508del-CFTR had reduced R_t_ values compared to those expressing wild-type CFTR, whereas Li et al. [32] found the converse. As with these previous studies, differences in the cells studied and the experimental conditions used likely explain the distinct results obtained with *FOXI1* KO airway epithelia.

We found that ALI cultured hiPSC-AECs without ionocytes show reduced cilia motility properties compared to cultures with ionocytes. More importantly, we showed that cultures without ionocytes displayed a smaller number of ciliated cells. This could be the reason for the slower movement of cilia in *FOXI1* KO cultures and it suggests that ionocytes could play a role in mucociliary clearance by influencing the production of ciliated cells. These results do not contradict the previous report by Montoro et al. showing that the absence of Foxi1 in mice led to more viscous mucous secretions in the airway epithelium and, in turn, higher CBF [2]. Our ALI cultured hiPSC-AECs do not produce abundant mucus, and CBF measurements did not change after washing the epithelia with PBS. Therefore, we cannot exclude the possibility that the absence of *FOXI1* expression could also increase mucus viscosity. However, it could be interesting to confirm if the number of ciliated cells is also decreased in a mouse KO for Foxi1. In the study by Goldfarbmuren et al. [6], *FOXI1* KO did not significantly affect *FOXJ1* mRNA expression, consistent with our RT-qPCR results. However, changes in ciliated cell numbers or expression of key markers at a protein level were not tested in their study. Importantly, our results are reinforced by studies in *Xenopus laevis* epidermis [33] which reported that the almost complete absence of Foxi1 led to a reduced number and aberrant morphology of cilia. Interestingly, engraftment of Foxi1 WT epidermis patches rescued the ciliation of the nearby KO epidermis. Thus, the importance of ionocytes in the production of ciliated cells could be conserved between species and tissues.

Various mechanisms could be driving the decrease of ciliated cells in the absence of FOXI1. First, the KO of *FOXI1* could be directly interfering with differentiation of ciliated cells. However, *FOXI1* is not expressed during the production of these cells [1, 2] and there is no evidence that the lineage of these two cell types is interdependent even if they both originate from basal cells [2, 6]. Second, cell-to-cell contact could be necessary between ionocytes and ciliated cells for the proper differentiation of the latter. This hypothesis would fit with the results obtained with *Xenopus laevis* epidermis [33]. Furthermore, ionocyte and ciliated cell differentiation is tightly controlled by Notch signalling. Thus, Notch-related crosstalk between the two cell types could play a role in the maturation of ciliated cells. Finally, it has been shown that ion channels and transporters highly expressed in ionocytes, such as the VATPase, are important in the regulation of Wnt signalling [34–36]. Interestingly, canonical Wnt/beta-catenin signalling has a role in the activation of the cilia development machinery via the regulation of *FOXJ1* expression [37–39], while the Wnt planar cell polarity signalling pathway is responsible for actin organisation and cilia beat alignment and coordination [40, 41]. The lack of ionocytes could affect the acidification of the microenvironment thereby blocking Wnt signalling and ciliated cell differentiation. Further investigation of AECs will help elucidate how such pathways can be controlled by pulmonary ionocytes.

## Conclusion

Overall, our study confirms that hiPSCs can be differentiated into an airway epithelium containing ionocytes and that *FOXI1* KO leads to a depletion of these cells. We show that the absence of ionocytes leads to impairment of epithelial barrier properties and ciliated cell homeostasis, revealing their potential role in the formation of the airway epithelium. This information represents an important step toward understanding the function of these cells in normal homeostasis and in lung disease, paving the way for new therapeutic applications focusing on ionocytes control.

### Electronic supplementary material

Below is the link to the electronic supplementary material.

Supplementary Material 1

Supplementary Material 2

Supplementary Material 3

## Data Availability

The single guide RNA and RT-qPCR primer sequences used for this study are provided in the Supplementary Materials and Methods. The code used to analyse cilia motility and coverage is publicly available [26] and the obtained data can be found in the Zenodo repository (DOI: 10.5281/zenodo.8309970). Other datasets and materials used and/or analysed in this study are available from the corresponding authors on reasonable request.

## Abbreviations

AcTub: acetylated tubulin
AEC: airway epithelial cell
ALI: air liquid interface
ASCL1: achaete-scute family bHLH transcription factor 1
ASCL3: achaete-scute family bHLH transcription factor 3
ASL: airway surface liquid
BSND: barttin CLCNK type accessory subunit beta
CBF: ciliary beat frequency
CF: cystic fibrosis
CFTR: cystic fibrosis transmembrane conductance regulator
CK5: cytokeratin 5
CPM: carboxypeptidase-M
CP110: centrosomal protein of 110 kDa
CRISPR: clustered regularly interspaced short palindromic repeats
DAPT: (2 S)-N-[2(3,5-Difluorophenyl)acetyl]-L-alanyl-2-phenyl-glycine 1,1-Dimethylethyl ester
DNAI1: dynein axonemal intermediate chain 1
DNAH5: dynein axonemal heavy chain 5
FGF10: fibroblast growth factor 10
FOV: field of view
FOXI1: forkhead box I1
FOXJ1: forkhead box J1
GCRP: calcitonin gene-related peptide
GFP: green fluorescent protein
hiPSC: human induced pluripotent stem cell
HBEC: human bronchial epithelial cell
hiPSC-AECs: human induced pluripotent stem cell-derived airway epithelial cells
HKG: housekeeping gene
I_SC_: short circuit current
KO: knock-out
mRNA: messenger RNA
MUC5AC: mucin 5AC
NEK10: NIMA related kinase 10
NKX2.1: NK2 homeobox 1
PBS: phosphate-buffered saline
PNEC: pulmonary neuroendocrine cell
R_t_: transepithelial resistance
RT-qPCR: reverse transcription-quantitative polymerase chain reaction
SAG: Smoothened agonist
SCGB3A2: secretoglobin family 3 A member 2
sgRNA: single guide RNA
SOX2: SRY-box transcription factor 2
STAP1: signal transducing adaptor family member 1
TP63: tumour protein p63
WT: wild-type
