# Supplementary material for: In vitro platform to model the function of ionocytes in the human airway epithelium
Source: Respir Res. 2024 Apr 25;25:180. doi: 10.1186/s12931-024-02800-7 (PMC11045446; doi:10.1186/s12931-024-02800-7)
Supplement: Supplementary file 1 — Supplementary Material 1 [file 12931_2024_2800_MOESM1_ESM.pdf]

SUPPLEMENTARY MATERIALS AND METHODS

hiPSC culture and differentiation to lung progenitors

hiPSCs were cultured on vitronectin XF™ (10 µg/ml, STEMCELL Technologies)-coated plates in chemically defined E8 medium as previously described [1]. hiPSCs were subjected to endoderm differentiation by dissociating them in StemPro™ Accutase™ Cell Dissociation Reagent (A1110501, Gibco) and seeding them at a density of 25000-100000 cells/cm^2 on gelatin/MEF (mouse embryonic fibroblast) medium coated plates in chemically defined E8 maintenance medium + 10 µM Y-27632. 24 hours after seeding, medium was replaced by E8 maintenance medium without Y-27632. On day 1 of differentiation, cells were fed with CDM-PVA (chemically defined medium with polyvinyl alcohol) medium supplemented with 80 ng/ml FGF2 (Dr. Marko Hyvönen, Cambridge University), 100 ng/ml Activin A (Dr. Marko Hyvönen, Cambridge University), 10 µM LY294002 (Promega), 10 ng/ml bone morphogenetic protein 4 (BMP4; Bio-Techne), and 3 µM CHIR-99021 (Tocris Bioscience). On day 2, the same medium without CHIR-99021 was used. On day 3, cells were fed with RPMI-B27 media supplemented with 80 ng/ml FGF2 and 100 ng/ml Activin A. At day 4, when cells had reached the definitive endoderm stage, cells were cultured in Basal Medium (composed of Advanced DMEM/F12 (12634010, Gibco) supplemented with L-ascorbic acid (LAA) 0.05 mg/ml, Monothioglycerol 0.4 mM, Glutamax™ 1X, Penicillin/Streptomycin 1% v/v and B27 1X) with 10 µM SB431542 and 100 ng/ml Noggin for 4 days. From day 9 to day 15, cells were cultured in Basal Medium with 3 µM CHIR-99021, 20 ng/ml BMP4 and 50-100 nM retinoic acid (50 nM for FS13B cells and 100 nM for CF17/NKX2.1-GFP cells). Medium was replaced daily. After 16 days of differentiation, hiPSC-derived lung progenitors were sorted.

Fluorescence activated cell sorting (FACS)

To detach cells from wells, they were washed with PBS and incubated with TrypLE™ Express Enzyme (12604039, Thermo Fisher) for 15 minutes at 37° C. The cells were further dissociated by gently pipetting the cell solution. After the neutralisation of TrypLE™ Express Enzyme by addition of one volume of Basal Medium, cells were counted and the desired amount of cells placed into new conical tubes through a 40 µm cell strainer. Cells were collected by centrifugation at 200 gx for 5 minutes at room temperature (RT) and the pellets were resuspended at a concentration of 10 million cells per ml in FACS buffer (Hank's Balanced Salt solution (HyClone) with FBS 2% v/v, Penicillin/Streptomycin 1% v/v, HEPES 25 mM and EDTA 2 mM) with Y-27632 10 µM (FACS+Y buffer). Cells were stained with PerCP/Cyanine5.5 anti-human CD47 antibody (323110, Biolegend) and/or PE anti-human CD26 antibody (302706, Biolegend) at a concentration of 5 µl/ml of cell solution for 30 minutes at 4 °C in the dark. After a wash with FACS+Y buffer, cells were collected by centrifugation at 200 gx for 5 minutes at RT and resuspended in FACS+Y buffer at a concentration of 10 million cells/ml. CD26^{lo}CD47^{hi} cells were sorted into a 15 ml tube with 1% bovine serum albumin (BSA) in PBS solution using a BD Influx™ Cell Sorter (BD Biosciences). Alternatively, anti-carboxypeptisase M (CPM) antibody (Wako FUJIFILM) was used to sort CPM+ cells [2]. CF17/NKX2.1-GFP reporter cells were purified by sorting NKX2.1:GFP+ cells [3].

Organoid culture

Sorted cells were collected by centrifugation at 300 gx for 3 minutes at RT and the pellets were resuspended at a concentration of 1000 cells/µl in Expansion Medium (Basal Medium supplemented with 3 µM CHIR-99021, 100 ng/ml FGF10 and 10 µM Y-27632). Cells were seeded in droplets of 10 µl of cell solution mixed with 10 µl of Matrigel® Growth Factor Reduced (GFR) basement membrane matrix (356231, Corning) in the centre of the wells of a 48 well plate. Organoid domes were incubated at 37 °C for 30 minutes to allow polymerisation and then 200 µl of organoid expansion medium were added. The medium was replaced the next day and then every 2-3 days for 10-14 days. From day 10, organoids could be cryopreserved

in CellBanker2 medium (11891, AMSBIO), expanded or seeded for air-liquid interface (ALI) culture.

To passage organoids, wells were washed with PBS and 400 µl TrypLE™ Express Enzyme were added per well. Domes were disrupted with a sterile pipette tip and cell solution was transferred to a 15 ml conical tube and incubated at 37 °C for up to 20 minutes. After further dissociation via gentle pipetting, cells were collected by centrifugation at 300 gx for 3 minutes at RT. Supernatant was discarded and, after one wash with Basal Medium, cells were resuspended in 1:1 Expansion Medium and Matrigel® GFR at 500 cells/µl. Domes of 20 µl were seeded and, after polymerisation for 30 minutes at 37°C, 200 µl of Expansion Medium were added per well. Medium was refreshed every 2-3 days.

ALI culture

Corning® Transwell® polyester membrane cell culture inserts (CLS3460, Corning) were coated with Matrigel® GFR at a concentration of 115 µg/ml and incubated for at least one hour at 37 ºC prior to cell seeding. Next, Matrigel® GFR was removed and replaced with Maturation Medium (Basal Medium with FGF7 10 ng/ml, FGF10 10 ng/ml, cAMP 0.1 mM, 3-isobutyl-1-methylxanthine (IBMX) 0.1 mM, dexamethasone 50 nM and Y-27632 10 µM), 0.5 ml in the insert and 1 ml in the underlying well. To collect lung progenitors in 3D organoid cultures to seed ALI cultures, the medium was removed from the wells and, after one wash with PBS, organoids were collected with 400 µl TrypLE™ Express Enzyme per Matrigel® GFR dome and mechanically dissociated by gently pipetting up and down inside a 15 ml tube before incubating the cell solution at 37 °C for 10 minutes. After further gentle pipetting to ensure cellular dissociation, 1 volume of medium was added to the solution. After cell counting, the desired number of cells was centrifuged at 200 gx for 5 minutes at RT. The supernatant was removed and the pellet was resuspended with Maturation Medium at a concentration of 800000 cells/ml. 400000 cells were seeded per 12 mm Transwell® insert (for 6.5 mm inserts, 250000 cells were seeded). Maturation Medium was refreshed the next day. Once the cells reached confluency, the medium in the top chamber (bathing the apical membrane) was carefully removed and Maturation Medium with the γ-secretase inhibitor DAPT (10 µM) was added to the bottom chamber to create an ALI culture. For increased ciliation, Maturation Medium + DAPT was replaced by a 1:1 mixture of PneumaCult™-ALI Medium (#05001, STEMCELL Technologies) and Maturation Medium + DAPT on day 14 after initiating ALI culture (when ciliated cells started to appear) and by PneumaCult™-ALI Medium after that. Cultures were kept for 28 days, changing the medium in the basolateral chamber every 2-3 days prior to study.

Culture of BMI1-transduced human bronchial epithelial cell culture

Normal human bronchial epithelial (NHBE) cells, also referred to as HBECs, used for the validation of immunofluorescent staining of ionocyte markers were purchased from the Primary Airway Cell Biobank at McGill University and transduced by lentivirus with *BMI-1* at MOI-4 as previously described [4]. Cells were cultured in PneumaCult™ Ex-Plus and matured at an ALI in PneumaCult™-ALI medium in Transwell® inserts as described above for hiPSC-AEC cultures.

RNA extraction, retrotranscriptase PCR and quantitative PCR (qPCR)

Total RNA was extracted by using GenElute™ Mammalian Total RNA Miniprep Kit (Sigma-Aldrich) together with the On-Column DNase I Digestion set (Sigma-Aldrich) following the manufacturer's instructions. 500 ng cDNA were synthesised from purified RNA by reverse transcription using Random Primers and SuperScript™ II (Invitrogen) according to

manufacturer's instructions. The resulting cDNA was diluted to a concentration of 0.83 ng/µl. For qPCR, each reaction contained 2.5 ng cDNA in 3 µl, 5 µl KAPA SYBR® FAST qPCR Master Mix (2X) (Kapa Biosystems), 0.4 µl of each primer (final concentration 200 nM) and 1.2 µl nuclease-free water. Reactions were performed in a 384 well plate using the QuantStudio 12K Flex Real-Time PCR System (Thermo Fisher Scientific). Results were normalised to three reference genes (ubiquitin C (*UBC*), acidic ribosomal phosphoprotein P0 (*RPLP0*) and porphobilinogen deaminase (*PBGD*), which were selected because their combination has proven to provide a highly stable reference across different cell types) and processed using the delta-delta cycle threshold method ($2^{-\Delta\Delta CT}$). Primer sequences are listed in Table S1.

Table S1: qPCR primer sequences.

Target	Forward primer (5'-3')	Reverse primer (5'-3')
UBC	ATTTGGGTCGCGGTTCTTG	TGCCTTGACATTCTCGATGGT
RPLP0	GGCGTCCTCGTGGAAGTGAC	GCCTTGCGCATCATGGTGTT
PBGD	GGAGCCATGTCTGGTAACGG	CCACGCGAATCACTCTCATCT
OCT4	AGTGAGAGGCAACCTGGAGA	ACACTCGGACCACATCCTTC
SOX17	CGCACGGAATTTGAACAGTA	GGATCAGGGACCTGTCACAC
FOXA2	GGGAGCGGTGAAGATGGA	TCATGTTGCTCACGGAGGAGTA
NKX2.1	CGGCATGAACATGAGCGGCAT	GCCGACAGGTACTTCTGTTGCTTG
SOX2	TGGACAGTTACGCGCACAT	CGAGTAGGACATGCTGTAGGT
TP63	CCACCTGGACGTATTCCACTG	TCGAATCAAATGACTAGGAGGGG
CFTR	CTATGACCCGGATAACAAGGAGG	CAAAAATGGCTGGGTGTAGGA
MUC5AC	GCACCAACGACAGGAAGGATGAG	CACGTTCCAGAGCCGGACAT
SCGB3A2	CCTTGTGGAGGGGCTAAGGA	ACACCAAGTGTGATAGCGCCT
FOXJ1	GAGCGGCGCTTTCAAGAAG	GGCCTCGGTATTCACCGTC
CDX2	GGCAGCCAAGTGAAAACCAG	TTCCTCTCCTTTGCTCTGCG
HNF4	CATGGCCAAGATTGACAACCT	TTCCCATATGTTCCTGCATCAG
NEK10	AGCCTGTCCAGTGCAAATG	GGCATTGGTGTCATTGAGC
DNAH5	AGGGCAGACACTCTAGGAGAA	GAGAGCCAAGTTGTCCTGTTTC
CP110	CTTAGTAGACAAGGAACCCC	CCGCTTTCTTTGGATTTTTC

Immunofluorescent staining

Medium was removed from the wells and cells were washed once with cold PBS before being fixed with 4% (w/v) paraformaldehyde (043368.9M, Thermo Fisher Scientific) for 20 minutes at 4 °C. Next, after 2 washes with PBS, cells were blocked and permeabilised for 30 minutes at RT with 10% donkey serum (Bio-Rad) and 0.1% Triton X-100 (Sigma-Aldrich) in PBS. Primary antibodies (Table S2) were diluted in PBS containing 1% donkey serum and 0.1% Triton X-100. Cells were incubated with primary antibody solutions at 4 °C overnight. After three 5-minute washes with PBS, cells were incubated with secondary antibodies (Table S3) diluted 1:1000 in PBS containing 1% donkey serum and 0.1% Triton X-100 for 1 hour at RT in the dark. Following a 5-minute wash with PBS, cells were treated with Hoechst stain diluted 1:10000 in PBS for 5 minutes at RT to identify nuclei. Following two 5-minute washes, cells were either kept in PBS or mounted in ProLong™ Gold Antifade Mountant (P36934, Thermo Fisher Scientific).

For organoid staining, fixation was performed at RT for 30 minutes, blocking lasted 1 hour, antibody incubations were performed at 4 °C overnight and washes were 45 minutes in duration. For the staining of hiPSC-AEC ALI cultures, Transwell® membranes were cut into pieces after the blocking step, stained in a 48-well plate and then mounted between a glass slide and a coverslip using ProLong™ Gold Antifade Mountant. For the staining of histological sections of hiPSC-AEC ALI cultures, epithelial membranes were cut out of the Transwell® insert and placed in a cassette for dehydration and paraffinization. After embedding and

cutting, sections were stained and mounted on slides in ProLong™ Gold Antifade Mountant. Imaging was performed using a Zeiss confocal microscope.

Table S2: Primary antibodies used for immunofluorescent staining.

Target	Application	Dilution	Reference number	Company
NKX2.1	IF	1:200	ab76013	abcam
SOX2	IF	1:50	AF2018	R&D
CK5	IF/IHC	1:200	ab17130	abcam
TP63	IF	1:200	ab124762	abcam
CFTR	IF	1:200	ab2784	abcam
FOXI1	IF	1:200	ab20454	abcam
MUC5AC	IF	1:200	MA1-38223	Thermo Fisher Scientific
AcTub	IF/IHC	1:400	#5335	CST
SCGB3A2	IF	1:500	ab181853	abcam
ASCL1	IF	1:100	ab211327	abcam
CGRP	IF	1:50	ab81887	abcam
GFP	IF	1:1000	ab13970	abcam
CK5 (mouse)	IF	1:1000	905901	Biolegend

Table S3: Secondary antibodies used for immunofluorescent staining.

Antibody	Dilution	Reference number	Company
Anti-mouse AF488	1:1000	A-21202	Life Technologies
Anti-mouse AF586	1:1000	A-10037	Life Technologies
Anti-mouse AF647	1:1000	A-31571	Life Technologies
Anti-goat AF488	1:1000	A-11055	Life Technologies
Anti-goat AF568	1:1000	A-11057	Life Technologies
Anti-goat AF647	1:1000	A-21447	Life Technologies
Anti-rabbit AF488	1:1000	A-21206	Life Technologies
Anti-rabbit AF586	1:1000	A-10042	Life Technologies
Anti-rabbit AF647	1:1000	A-31573	Life Technologies
Anti-chick AF488	1:1000	A-11039	Life Technologies

Transepithelial resistance measurement

Transepithelial resistance (R_t) of hiPSC-AEC epithelia grown at an ALI was measured using an Epithelial Volt/Ohm Meter (EVOM2, World Precision Instruments) according to manufacturer's instructions after 200 µl of PneumaCult™-ALI medium were added to the apical chamber of the Transwell®. A Matrigel® GFR coated Transwell® bathed in the same medium was used as a blank. Readings were taken from 3 different points per Transwell®. Data from 6 hiPSC-AEC epithelia from 3 independent experiments were used for each condition.

Analysis of ciliary dynamics

High-speed videos of 20 arbitrary fields of view (FOV) across each hiPSC-AEC epithelium grown at an ALI were recorded while keeping the Transwell® inserts at 37 °C in a controlled atmosphere with 100% humidity and 5% CO_2 in a custom-made chamber designed and manufactured by Dr Jurij Kotar (Cavendish Laboratory, Department of Physics, University of

Cambridge). Imaging was repeated after removing the mucus layer by incubating the apical surface of the hiPSC-AEC epithelia in 200 µl of sterile PBS for 20 minutes at 37 ºC, and then removing the solution. Because our hiPSC-AEC ALI cultures are not covered in mucus and can appear dehydrated, the movement of cilia was better detected after washing them with PBS. Thus, only data from PBS-washed hiPSC-AEC epithelia have been included.

Each video was analysed with a robust Fourier Transform method. In brief, each video was first normalised so that its grey levels fell within a 0-1 range. The average frame was calculated as the mean grey value for each pixel over the entire analysis time and was then subtracted from the normalised video to remove the background. This was followed by a Power Spectral Density estimate over the normalised frame stack, resulting in a power spectrum for each individual pixel in the FOV. A 2-30 Hz band pass filter was applied at this point.

To isolate the areas of the hiPSC-AEC epithelia with moving cilia, the analysis relies on the evaluation of local changes to grey value frequency and intensity over different lengths. 15 different box sizes were first selected (5 to 33 pixels). For each box size, the Power Spectral Density estimate for each pixel was calculated as the average of the power spectrum of all pixels within the box around it (e.g. for a box size of 5 pixels, the new spectrum for a particular pixel is the average spectrum of a 5x5 pixel square, centred around itself). A linear regression was applied to each pixel spectrum, and the slope of the regression line was stored, effectively creating a map of the slopes of the spectrum of each pixel for that particular box size. A second linear regression was applied to the slope values for each pixel over the different box sizes, to create an approximated map of the changes in frequency and intensity over the entire FOV. To remove any small artefact of the analysis, a 5-pixel box average filter was applied to this map, and a background map was created by thresholding the absolute value of this approximation. In this way, the areas with cilia movement were isolated from the background.

To calculate the average ciliary beat frequency (CBF), a 3-pixel box average filter was applied to the original Power Spectral Density as described in the preceding paragraph, and each pixel frequency was calculated as the frequency corresponding to the highest amplitude local maxima. The CBF distribution was then calculated by single FOV or by sample over all of the measured FOVs. In some specific cases, a few data points were excluded from the cilia coverage data: videos of poor quality, videos with no movement where coverage values were >2% and videos that showed movement of debris or particles that interfered with the accuracy of the measurement (Figure S4). For CBF, only data from FOVs with >5% coverage that were included in the cilia coverage data were considered.

In vivo transplantation experiments

These experiments were approved by local ethical review committees and conducted according to Home Office project license PPL PEEE9B8E4 (Emma L. Rawlins, University of Cambridge).

Mice were weighed and anesthetised with 4% isofluorane for up to 4 minutes, injected subcutaneously with buprenorphine (0.03 mg/ml per 10 g body weight) and then further anesthetised with 4% isofluorane for 2 additional minutes. Then, mice were administered 30 µl of 2% polidocanol (Thesit®, 88315, Sigma-Aldrich) w/v in 1x PBS oropharyngeally through controlled aspiration. Next, they were put in the recovery cage and provided with a heat pad and Nutella and returned to their usual holding room overnight.

The following day, GFP+ hiPSC-derived lung progenitor organoids in Expansion Medium were collected in TrypLE™ Express Enzyme as detailed in the Organoid culture section, transferred into a 15 ml concical tube and incubated for 15 minutes at 37 °C. Cells were further mechanically dissociated by pipetting and counted in preparation to transplant 1 million cells into each mouse. Cells were centrifuged at 200 gx for 5 minutes at RT and resuspended in sterile PBS containing 0.1% BSA at a concentration of 33.3 million cells/ml. Aliquots of 30 µl

(1 million cells) were transferred into 1.5 ml tubes and kept on ice until the moment of transplantation. For cell transplantation, mice were anesthetised with 4% isofluorane and 30 µl of cell suspension were administered oropharyngeally through controlled aspiration. Mice were put back in the recovery cage.

A total of 9 mice were transplanted with hiPSC-derived lung progenitors. At different time points after transplantation (day 1 n = 3, day 7 n = 5, and day 10 n = 1), mice were sacrificed, tracheas dissected and whole mount immunofluorescence staining performed to visualise cells.

In brief, tracheas were fixed overnight in 4% (w/v) paraformaldehyde at 4 °C before being incubated for 3 days with primary antibodies anti-GFP (1:1000, ab13970, abcam), anti-human CK5 (1:200, ab17130, abcam) and/or anti-mouse CK5 (1:1000, 905901, Biolegend). After 3 washes, tracheas were incubated with secondary antibodies anti-chick AF488 (1:1000, A-11039, Life Technologies) and anti-mouse AF568 (1:1000, A-10037, Life Technologies). Tracheas were mounted in ProLong™ Gold Antifade Mountant (P36934, Thermo Fisher Scientific) and sealed with nail polish before being imaged using a Zeiss 700 confocal microscope. Numbers of GFP+ and CK5+ cells were quantified with the Cell Counter plugin of ImageJ2 version 2.14.0/1.54f.

CRISPR/Cas9-based gene editing

Table S4 shows the sequences of the single guide RNAs (sgRNAs) designed to target the DNA binding domain of *FOXI1* that were purchased from SYNTHEGO. sgRNAs were rehydrated with Tris-EDTA buffer provided by supplier and Cas9 protein was thawed on ice. RNaseZAP RNAse decontamination solution (R2020, Sigma-Aldrich) was used to clean the equipment and surfaces prior to commencing gene-editing.

Cas9 protein was assembled with sgRNA by combining 20 µg of recombinant SpCas9 protein and 2.25 µl full length sgRNA (100 µM) in a sterile 1.5 ml tube. The mix was incubated for 10 minutes at RT and then stored on ice.

At least $1x10^6$ hiPSCs were collected by incubating them with StemPro™ Accutase™ Cell Dissociation Reagent for 3 minutes at 37 ºC and made into a single cell suspension. After Accutase was neutralised with TeSR™-E8™ human pluripotent stem cell medium (#05990, STEMCELL Technologies) medium with 10 µM Y-27632, cells were transferred to a conical tube and counted. $1x10^6$ cells were transferred to a new tube and pelleted by centrifuging at 120 gx for 3 minutes at RT. The supernatant was discarded and the pellet was washed with PBS. After another centrifugation at 120 gx for 3 minutes at RT, supernatant was discarded and cell pellet was resuspended in 100 µl of Amaxa P3 Primary Cell 4D-Nucleofector X Kit L solution (V4XP-3024, Lonza) and mixed with the Cas9-sgRNA solution. The mixture was transferred into an Amaxa 4D Nucleofector single cuvette avoiding air bubbles. After electroporation using Amaxa Nucleofector (Lonza), cells were collected using a soft plastic pipette and seeded dropwise into 3 wells of a six well plate previously coated with Vitronectin and filled with TeSR™-E8™ human pluripotent stem cell medium supplemented with 10 µM Y-27632. 24 hours after electroporation, the medium was changed to TeSR™-E8™ human pluripotent stem cell medium without Y-27632. Cells were grown for 3-7 days to reach 70% confluency. Once confluent, cells from different wells were genotyped using Titanium Taq DNA kit (639208, TaKaRa), cryopreserved and subcloned. After subcloned cells had formed colonies, a number of these were individually picked and further subcloned. Each of the subclones was genotyped and clones of interest (one wild-type and one homozygous knock-out for each genetic background) were expanded and used for *FOXI*1 KO experiments.

Table S4: Sequences of the single guide RNAs used for the targeting of the DNA binding domain of FOXI1.

Targeting strategy	Targeted cell line	Sequence (PAM sequence in bold)
#1	FS13B	GGCGCTTGTCGGGTGCCCCG**TGG**
#2	CF17/NKX2.1-GFP	GGAAGTTGTCGGCCACGTAC**TGG**

Western blotting

Cells were collected in PBS and centrifuged at 900 gx for 3 minutes at RT. Cell pellets were lysed in 100 µl of radioimmunoprecipitation assay (RIPA) buffer (Roche) with 1X PhosSTOP phosphatase inhibitor (4906845001, Roche) and 1X cOmplete protease inhibitor (5892791001, Roche) and incubated for 15 minutes at RT. Cell lysates were centrifuged at 20000 gx for 10 minutes at 4 ºC. The supernatants were transferred into a new tube and the amount of protein was quantified using a bicinchoninic acid (BCA) assay (Pierce) according to the manufacturer's instructions, using a standard curve based on BSA dilutions and spectrophotometer absorbance readings at 600 nm using an EnVision 2104 plate reader. Next, 4X NuPAGE™ LDS sample buffer (Thermo Fisher Scientific) with 4% 2-mercaptoethanol was added to the samples for a 1X final concentration.

20-50 µg of protein were separated using NuPAGE™ Bis-Tris Gel (10% for FOXI1 detection, 4-12% for DNAI1 detection, Thermo Fisher Scientific) and transferred to polyvinylidene difluoride (PVDF) membranes. Membranes were blocked for 1 hour at RT. 4% milk in PBS with 0.1% Tween-20 (PBS-T) was used as blocking buffer for detection of Vinculin and DNAI1. 1% milk in Tris-buffered saline (TBS) with 0.1% Tween-20 (TBS-T) was used for FOXI1 detection. Membranes were incubated overnight at 4 ºC with primary antibodies (Table S5) diluted in the corresponding blocking buffer. After 3 washes with either PBS-T or TBS-T, membranes were incubated with secondary HRP (horseradish peroxidase)-conjugated antibodies (Table S5) diluted in the corresponding blocking buffers for 1 hour at RT. After 3 more washes, membranes were incubated with Pierce™ ECL Plus Western Blotting Substrate (32134, Thermo Fisher Scientific) for 4 minutes at RT and exposed to an X-Ray film that was subsequently developed. Relative protein levels were quantified by measuring band intensities and normalising to corresponding controls using ImageJ2 version 2.14.0/1.54f.

Table S5: Primary and secondary antibodies used for Western blotting

Antibody	Dilution	Reference number	Company
Rabbit anti-DNAI1	1:500	HPA021649	Atlas antibodies
Rabbit anti-FOXI1	1:500	HPA071469	Atlas antibodies
Mouse anti-vinculin	1:1000	SAB4200080	Sigma-Aldrich
Anti-mouse HRP	1:5000	A2554	Sigma-Aldrich
Anti-rabbit HRP	1:5000	A0545	Sigma-Aldrich

pH measurement

On day 28 of ALI culture, 200 µl of PneumaCult™-ALI medium was added to the apical side of hiPSC-AEC epithelia. After a 2-hour incubation at 37 °C in a humidified atmosphere of 5% CO_2, 10 µl of medium bathing the apical membrane were transferred into a 500 µl tube and pH was measured within 2 minutes with an Orion™ 9810BN Micro pH Electrode (Thermo Fisher Scientific).

Flow cytometry

hiPSC-AECs in epithelia cultured at an ALI for 28 days were dissociated as detailed above (see "Fluorescence activated cell sorting (FACS)") and fixed with 4% (w/v) paraformaldehyde for 10 minutes at 4 ºC. After 2 washes with PBS containing 1% BSA, cells were blocked with PBS containing 10% FBS and 0.1% Saponin for 1 hour at RT. Next, cells were incubated with anti-FOXJ1 antibody (1:100, 14-9965-82, Thermo Fisher Scientific) for 30 minutes at RT. After 3 washes with PBS containing 1% BSA, cells were incubated with the fluorochrome-conjugated secondary antibody anti-mouse AF647 (1:1000, A-31571, Life Technologies) for 30 minutes at RT. After 3 more washes with PBS containing 1% BSA, cells were analysed with a BD Fortessa flow cytometer and results were processed with FlowJo™ 10.8.1. Cells stained with secondary antibody only and stained hiPSCs were used as controls.

Ussing chamber experiments

Ussing chamber studies to investigate the ion transport properties of hiPSC-AEC epithelia were performed as previously described [5] with some modifications. To form well-differentiated, polarised epithelia, hiPSC-AECs were cultured at an ALI on Millicell® Standing Cell Culture Inserts (cat no. PIHP01250, Merck Life Science UK, Ltd.) for 28 days prior to study. As controls, we studied Fischer rat thyroid (FRT) epithelia stably expressing wild-type and F508del human CFTR [6], generously provided by LJV Galietta (Telethon Institute of Genetics and Medicine, Pozzuoli, Italy). FRT cells were cultured as previously described [7] without rescuing the plasma membrane expression of F508del-CFTR by either low temperature incubation or treatment with CFTR correctors. FRT cells were studied 5–7 days after seeding when they had formed electrically tight epithelia (R_t > 1 kΩ cm^2).

Polarised hiPSC-AEC and FRT epithelia were mounted in Ussing chambers (Warner Instrument Corp. Dual Channel Chamber, model U-2500, Harvard Apparatus Ltd., Edenbridge, UK) and a chloride concentration gradient was imposed across epithelia to magnify transepithelial ion transport. The solution bathing the basolateral membrane of epithelia contained (mM): 140 NaCl, 5 KCl, 0.3 K_2HPO_4, 0.44 KH_2PO_4, 1.3 $CaCl_2$, 0.5 $MgCl_2$, 10 HEPES and 4.2 $NaHCO_3$, adjusted to pH 7.2 with Tris. The solution bathing the apical membrane was identical to that of the basolateral solution with the exception that 133.3 mM Na gluconate + 2.5 mM NaCl + 5 mM K gluconate replaced 140 mM NaCl and 5 mM KCl, respectively, to create a transepithelial Cl^- concentration gradient (basolateral [Cl^-], 149 mM; apical [Cl^-], 14.8 mM). To compensate for calcium buffering by gluconate, 5.7 mM Ca^{2+} was used in the apical solution. All solutions were maintained at 37 °C and bubbled continuously with 5% CO_2/95% O_2 under low pressure to circulate solutions and mix test compounds.

To study transepithelial ion transport, voltage off-sets were cancelled, transepithelial voltage (referenced to the basolateral solution) was clamped at 0 mV and short-circuit current (I_{sc}) was recorded continuously using an epithelial voltage-clamp amplifier (Warner Instrument Corp. model EC-825, Harvard Apparatus Ltd.) digitising data as previously described [8]. After baseline I_{sc} stabilised following the mounting of epithelia in Ussing chambers, the following was added to the apical solution: (i) amiloride (100 μM) to inhibit I_{sc} mediated by the epithelial Na^+ channel (ENaC), (ii) forskolin (10 μM) and IBMX (100 μM) to activate CFTR-mediated I_{sc}, (iii) the CFTR inhibitor $CFTR_{inh}$-172 (10 μM) to inhibit CFTR-mediated I_{sc} and (iv) UTP (100 μM) to stimulate I_{sc} mediated by the Ca^{2+}-activated Cl^- channel TMEM16A; all small molecules were added sequentially and cumulatively to the apical solution. Under the experimental conditions employed, flow of current from the basolateral to the apical solution corresponds to Cl^- movement through open CFTR or Ca^{2+}-activated Cl^- channels and is shown as an upward deflection. The resistance of the filter and solutions, in the absence of cells, was subtracted from all measurements. For illustration purposes, I_{sc} time courses are displayed as ΔI_{sc} with the I_{sc} value immediately preceding forskolin and IBMX addition designated as 0 μA/cm^2; file sizes were compressed by 50-fold data reduction.

SUPPLEMENTARY REFERENCES

1. Tomaz RA, Zacharis ED, Bachinger F, Wurmser A, Yamamoto D, Petrus-Reurer S, et al. Generation of functional hepatocytes by forward programming with nuclear receptors. Elife. 2022;11.
2. Konishi S, Gotoh S, Tateishi K, Yamamoto Y, Korogi Y, Nagasaki T, et al. Directed Induction of Functional Multi-ciliated Cells in Proximal Airway Epithelial Spheroids from Human Pluripotent Stem Cells. Stem cell reports. 2016 Jan 12;6(1):18–25.
3. Hawkins FJ, Suzuki S, Beermann M Lou, Barillà C, Wang R, Villacorta-Martin C, et al. Derivation of Airway Basal Stem Cells from Human Pluripotent Stem Cells. Cell Stem Cell. 2021;28(1):79-95.e8.
4. Munye MM, Shoemark A, Hirst RA, Delhove JM, Sharp T V, McKay TR, et al. BMI-1 extends proliferative potential of human bronchial epithelial cells while retaining their mucociliary differentiation capacity. Am J Physiol - Lung Cell Mol Physiol. 2017;312(2):L258-67.
5. Xin M, Yiting W, Xiaomeng W, Wrennall JA, Rimington TL, Li H, et al. Two small molecules restore stability to a subpopulation of the cystic fibrosis transmembrane conductance regulator with the predominant disease-causing mutation. J Biol Chem. 2017;292(9):3706-19.
6. Sheppard DN, Carson MR, Ostedgaard LS, Denning GM, Welsh MJ. Expression of cystic fibrosis transmembrane conductance regulator in a model epithelium. Am J Physiol. 1994;266:L405-13.
7. Hughes LK, Ju M, Sheppard DN. Potentiation of cystic fibrosis transmembrane conductance regulator (CFTR) Cl− currents by the chemical solvent tetrahydrofuran. Mol Membr Biol. 2008;25(6–7):528–38.
8. Li H, Findlay IA, Sheppard DN. The relationship between cell proliferation, Cl− secretion, and renal cyst growth: A study using CFTR inhibitors. Kidney Int. 2004;66(5):1926–38.
