# Supplementary material for: In vitro platform to model the function of ionocytes in the human airway epithelium
Source: Respir Res. 2024 Apr 25;25:180. doi: 10.1186/s12931-024-02800-7 (PMC11045446; doi:10.1186/s12931-024-02800-7)

Full blots for Western blot images in Figure 4G

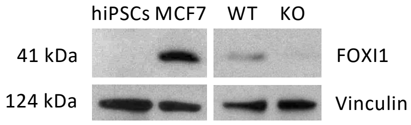

High exposure

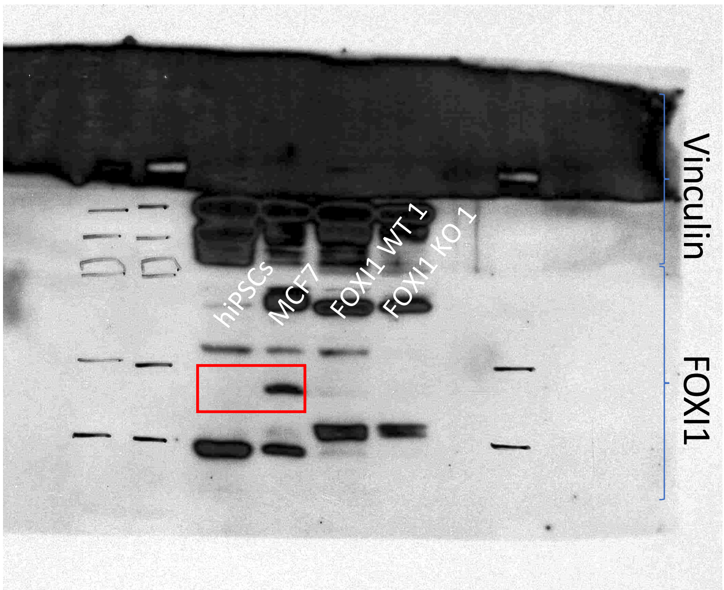

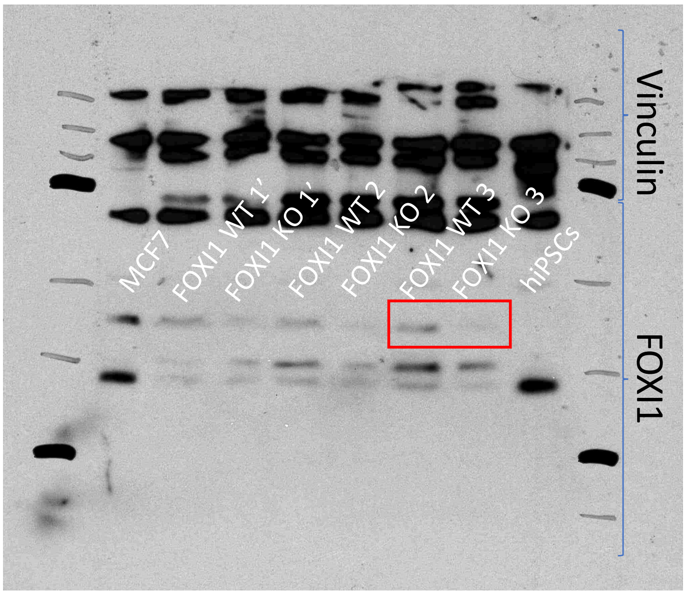

Low exposure

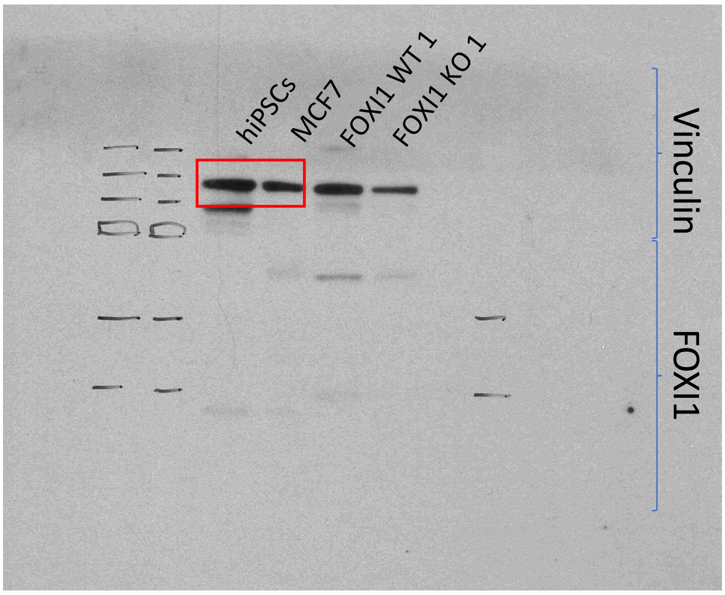

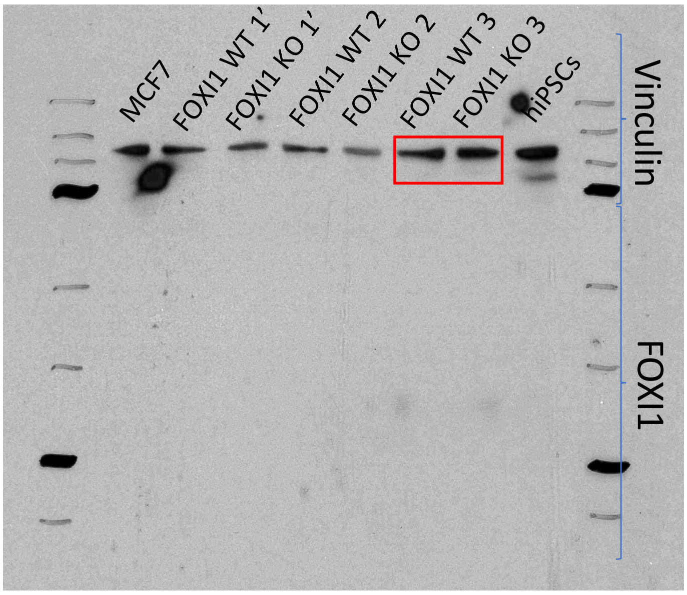

FOXI1 WT and KO 1 and 1' come from ALIs from the same differentiation, seeded from organoids at different passages

Full blots for Western blot images in Figure 5F

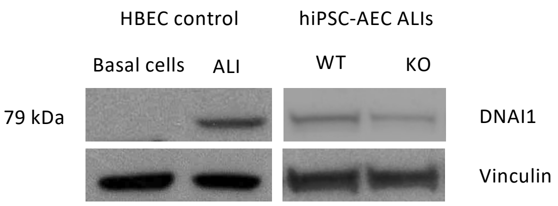

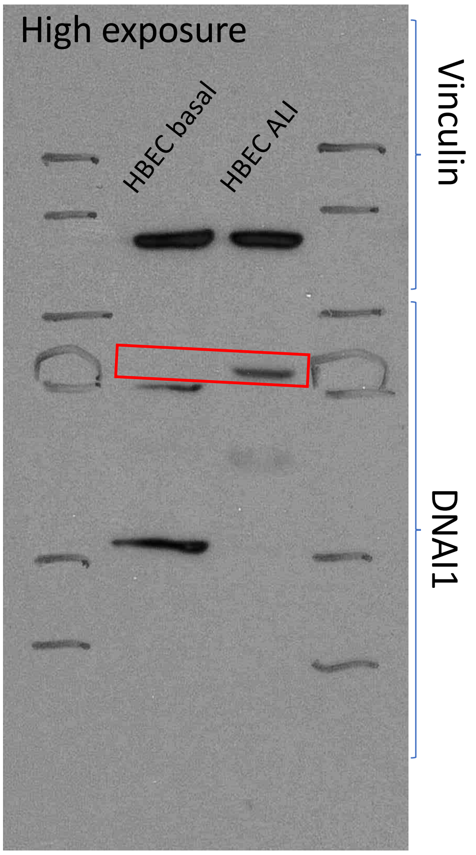

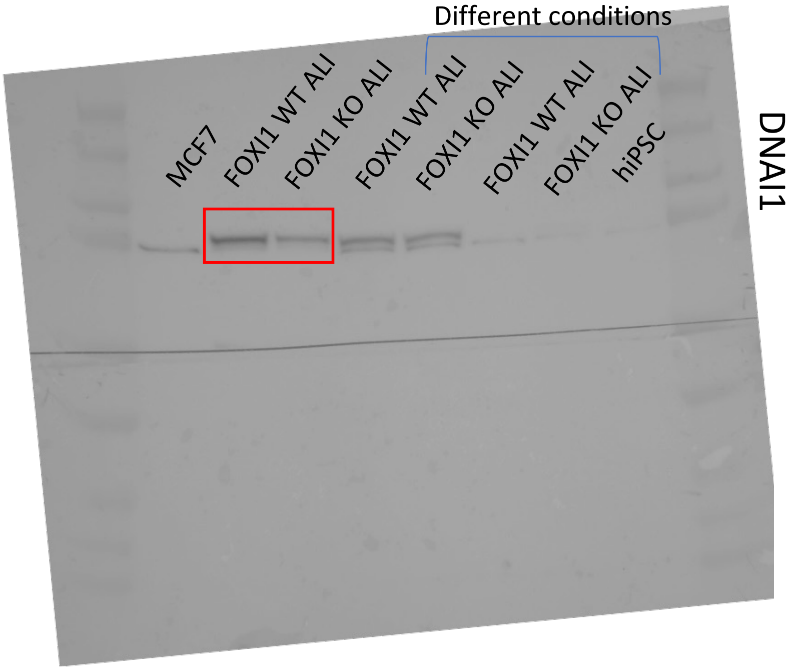

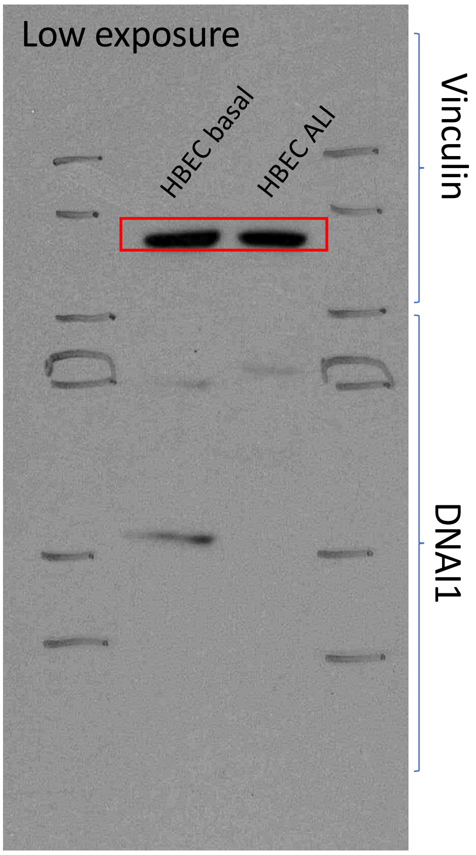

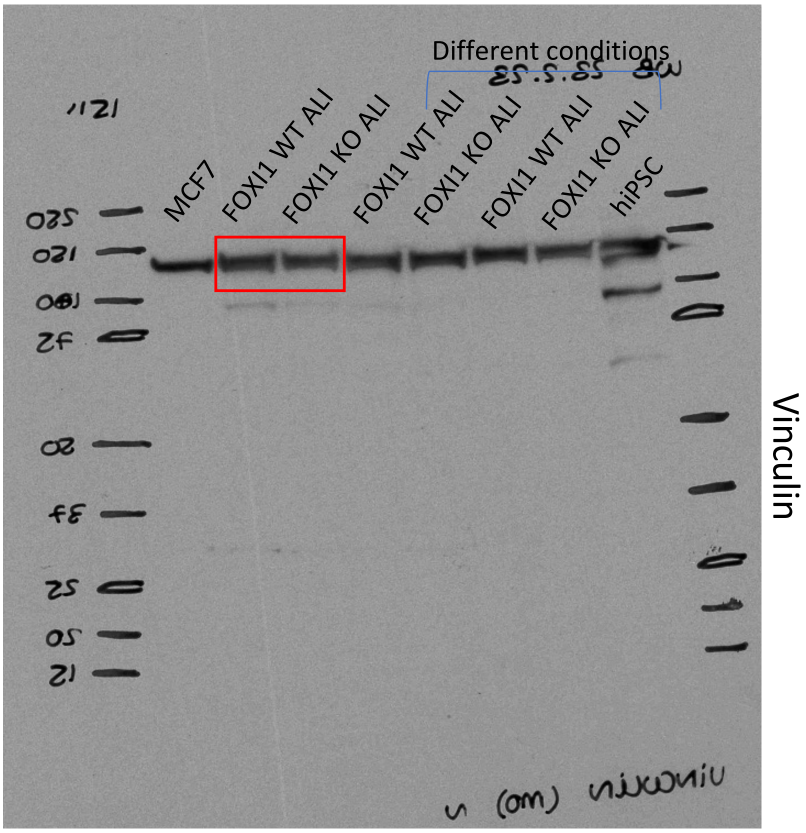

Supplement: Supplementary file 2 — Supplementary Material 2 [file 12931_2024_2800_MOESM2_ESM.pdf]
