# Supplementary material for: In vitro platform to model the function of ionocytes in the human airway epithelium
Source: Respir Res. 2024 Apr 25;25:180. doi: 10.1186/s12931-024-02800-7 (PMC11045446; doi:10.1186/s12931-024-02800-7)
Supplement: Supplementary file 3 — Supplementary Material 3 [file 12931_2024_2800_MOESM3_ESM.pdf]

SUPPLEMENTARY FIGURES

Figure S1

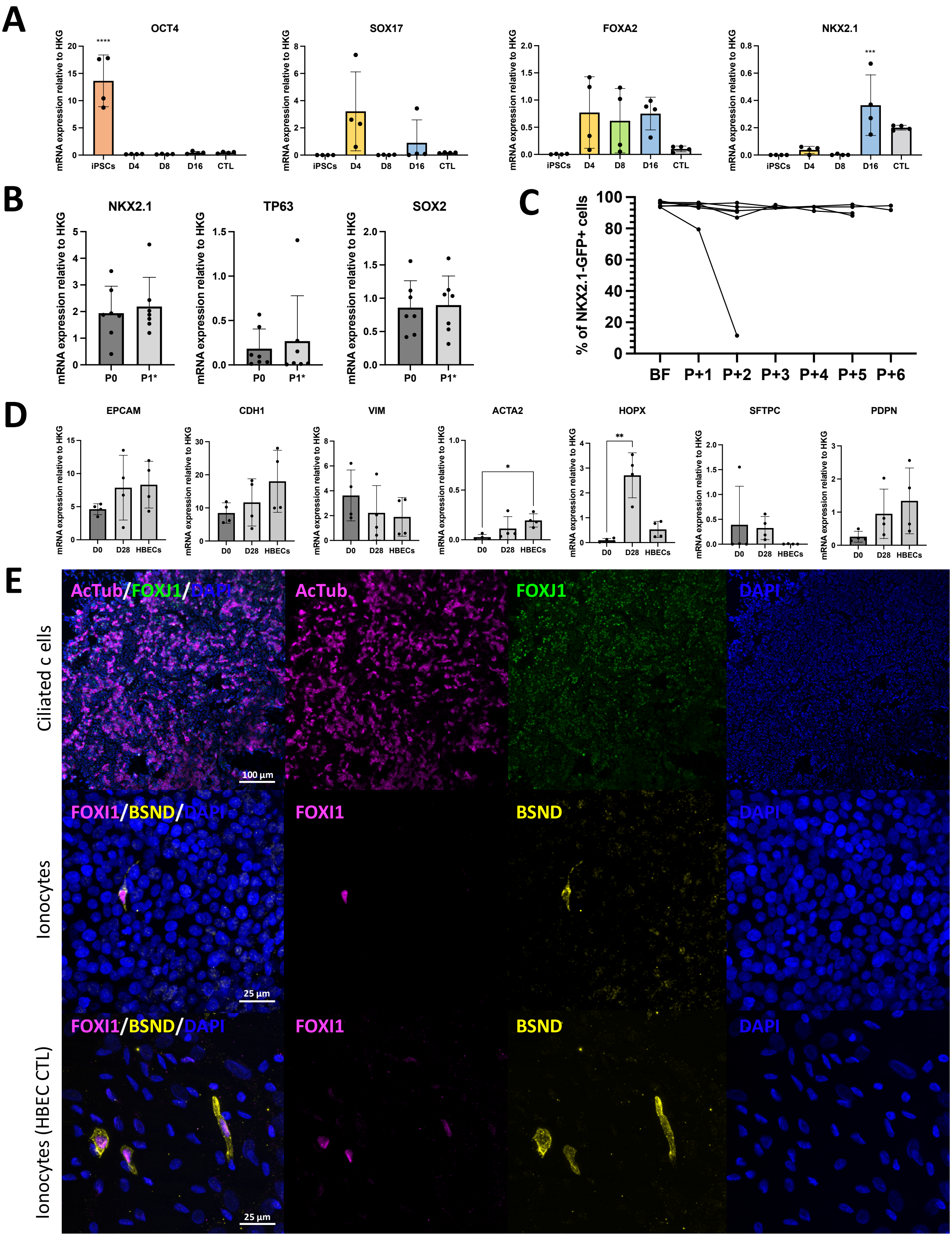

A
OCT4
SOX17
FOXA2
NKX2.1
mRNA expression relative to HKG
iPSCs
D4
D8
D16
CTL
B
NKX2.1
TP63
SOX2
P0
P1*
C
% of NKX2.1-GFP+ cells
BF
P+1
P+2
P+3
P+4
P+5
P+6
D
EPCAM
CDH1
VIM
ACTA2
HOPX
SFTPC
PDPN
D0
D28
HBECs
E
Ciliated c ells
Ionocytes
Ionocytes (HBEC CTL)
AcTub/FOXJ1/DAPI
AcTub
FOXJ1
DAPI
FOXI1/BSND/DAPI
FOXI1
BSND
100 µm
25 µm

Figure S1. Expression of key markers at different AEC differentiation stages. A: Relative mRNA expression of key markers at different time points of differentiation. The control (CTL) is human trachea total mRNA. Filled circles represent individual data points and columns are means ± SD (*n* = 4 independent experiments); ***P < 0.001, ****P < 0.0001, one-way ANOVA with Tukey's post-test. B: Relative mRNA expression of progenitor and basal cell markers in organoids in expansion medium at passage 0 (P0) and P1, after thawing (genetic background 1). Filled circles represent individual data points and columns are means ± SD (*n* = 7 independent experiments); not significant, Wilcoxon matched pairs signed ranks test. C: Percentage of NKX2.1-GFP expressing cells in organoids before freezing (BF) and after thawing through several passages. Filled circles represent individual data points. Points from the same experiment are connected. *N* = 5 independent experiments. D: Relative mRNA expression of various markers in airway progenitor organoids (D0) and hiPSC-AEC ALI cultures (D28). HBEC ALI cultures were used as controls. Filled circles represent individual data points and columns are means ± SD, *n* = 4 independent experiments. *P < 0.05, **P < 0.01, Kruskal-Wallis test with Dunn's post-test. E: Representative immunofluorescence staining of ciliated cells (AcTub (magenta) and FOXJ1 (green)) and ionocytes (BSND (yellow) and FOXI1 (magenta)) in CF17/NKX2.1-GFP hiPSC-AECs. Ionocytes in HBEC ALI cultures were used as a positive control. The scale bars are 100 μm and 25 μm.

Figure S2

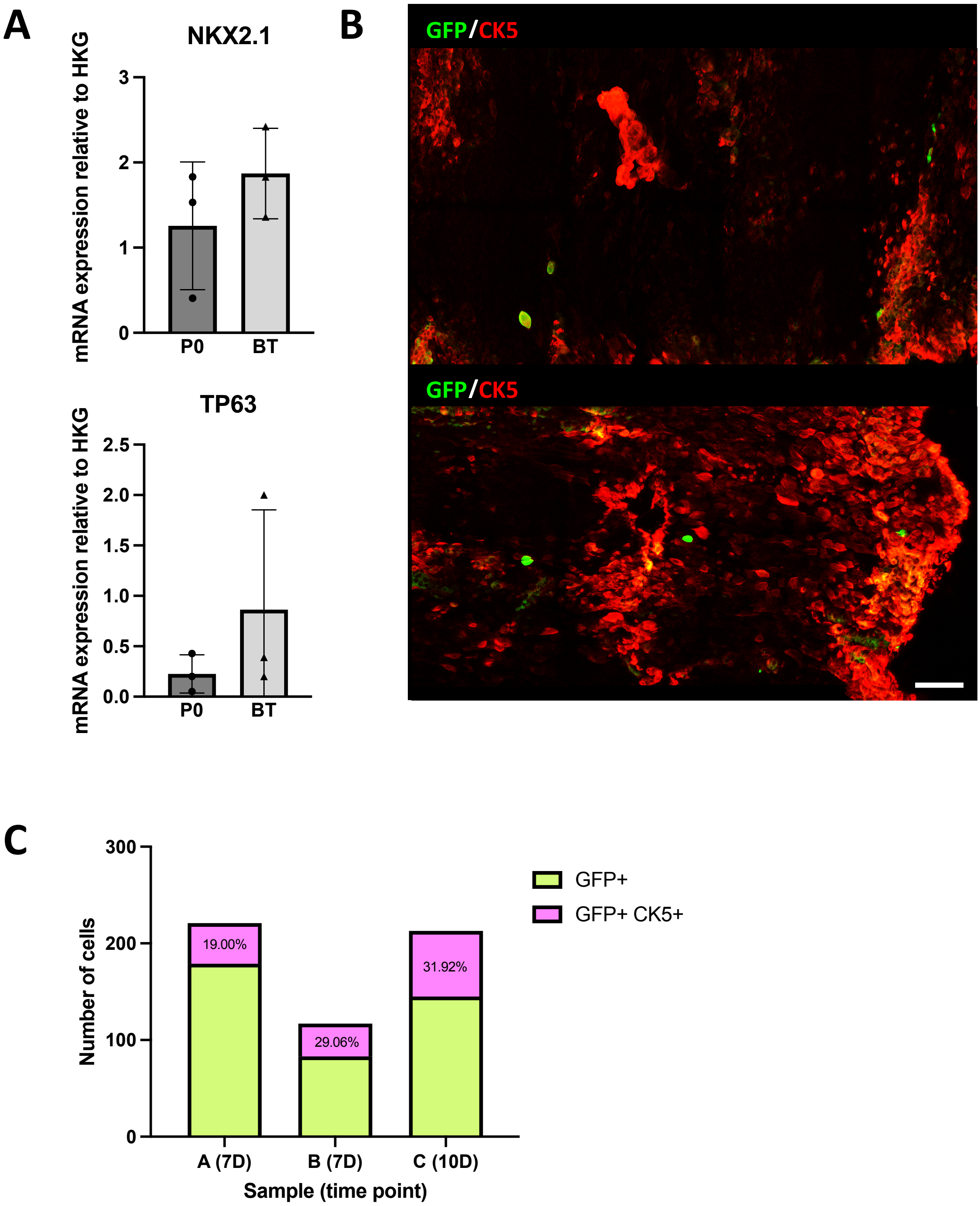

Figure S2. hiPSC-derived lung progenitors engraft in a murine model of airway injury. A: Relative mRNA expression of lung progenitor markers NKX2.1 and TP63 in organoids at P0 and before transplant (BT). Filled circles represent individual values and columns are means ± SD (n = 3 independent experiments); not significant, Student's t-test. B: Representative wholemount immunofluorescence staining shows GFP+ hiPSC-derived lung progenitors (green) and mouse CK5 (red) on day 1 (top) and day 7 (bottom) after cell transplantation. The scale bar is 100 µm. C: Graph representing the number of GFP+ hiPSC-derived cells that were present in whole mounted tracheas from 3 different mice (A, B and C). The bars represent the total of cells found per mice. In each, the pink section corresponds to GFP+ CK5+ counts and the green section to GFP+ CK5- cells. The percentages indicate the proportion of cells that co-expressed CK5 in the total number of cells.

Figure S3

A

WT

FOXI1 Transcript variant 1 (378 aa):

MSSFDLPAPSPPRCSPQFPSIGQEPPEMNLYYENFFHPQGVPSPQRPSFEGGGEYGATPNPYLWFNGPTMTPPPYLPGPNASPFLPQAYGVQRPLLPSVSGLGGSDLGWLPIPSQEELMKLV
RPPYSYSALIAMAIHGAPDKRLTLSQIYQYVADNFPFYNKSKAGWQNSIRHNLSLNDCFKKVPRDEDDPGKGNYWTLDPNCEKMFDNGNFRRKRKRKSDVSSSTASLALEKTESSLPVDSPKTT
EPQDILDGASPGGTTSSPEKRPSPPPSGAPCLNSFLSSMTAYVSGGSPTSHPLVTPGLSPEPSDKTGQNSLTFNSFSPLTNLSNHSGGGDWANPMPTNMLSYGG**SVLSQFSPHFYNSVNTSGV
LYPREGTEV**

FOXI1 Transcript variant 2 (283 aa):

MSSFDLPAPSPPRCSPQFPSIGQEPPEMNLYYENFFHPQGVPSPQRPSFEGGGEYGATPNPYLWFNGPTMTPPPYLPGPNASPFLPQAYGVQRPLLPSVSGLGGSDLGWLPIPSQEELMKLV
RPPYSYSALIAMAIHGAPDKRLTLSQIYQYVADNFPFYNKSKAGWQNSIRHNLSLNDCFKKVPRDEDDPAYVSGGSPTSHPLVTPGLSPEPSDKTGQNSLTFNSFSPLTNLSNHSGGGDWANP
MPTNMLSYGG**SVLSQFSPHFYNSVNTSGVLYPREGTEV**

FS13B Targeting strategy #1

FS13B Cl06 FOXI1 KO Transcript variant 1 prediction:

MSSFDLPAPSPPRCSPQFPSIGQEPPEMNLYYENFFHPQGVPSPQRPSFEGGGEYGATPNPYLWFNGPTMTPPPYLPGPNASPFLPQAYGVQRPLLPSVSGLGGSDLGWLPIPSQEELMKLV
RPPYSYSALIAMAIHGTRQAPHSQPDLPVRGRQLPLLQQEQGRLAELHPPQPVAQRLLQEGAPRRGRPGQRELLDPGPQL*

FS13B Cl06 FOXI1 KO Transcript variant 2 prediction:

MSSFDLPAPSPPRCSPQFPSIGQEPPEMNLYYENFFHPQGVPSPQRPSFEGGGEYGATPNPYLWFNGPTMTPPPYLPGPNASPFLPQAYGVQRPLLPSVSGLGGSDLGWLPIPSQEELMKLV
RPPYSYSALIAMAIHGTRQAPHSQPDLPVRGRQLPLLQQEQGRLAELHPPQPVAQRLLQEGAPRRGRPGLCERGEPHEPPLGHTRTEP*

CF17/NKX2.1-GFP Targeting strategy #2

CF17/NKX2.1-GFP Cl07 FOXI1 KO Transcript variant 1 prediction:

MSSFDLPAPSPPRCSPQFPSIGQEPPEMNLYYENFFHPQGVPSPQRPSFEGGGEYGATPNPYLWFNGPTMTPPPYLPGPNASPFLPQAYGVQRPLLPSVSGLGGSDLGWLPIPSQEELMKLV
RPPYSYSALIAMAIHGAPDKRLTLSQIYQLSWPTTSPSTTRARPAGRTPSATTCRSTTASRRCPATRTTRAKGITGPWTPTVRKCSTMEISAGKGRENQMFPLAQPPWP*

CF17/NKX2.1-GFP Cl07 FOXI1 KO Transcript variant 2 prediction:

MSSFDLPAPSPPRCSPQFPSIGQEPPEMNLYYENFFHPQGVPSPQRPSFEGGGEYGATPNPYLWFNGPTMTPPPYLPGPNASPFLPQAYGVQRPLLPSVSGLGGSDLGWLPIPSQEELMKLV
RPPYSYSALIAMAIHGAPDKRLTLSRQLPLLQQEQGRLAELHPPQPVAQRLLQEGAPRRGRPGQRELLDPGPQL*

B

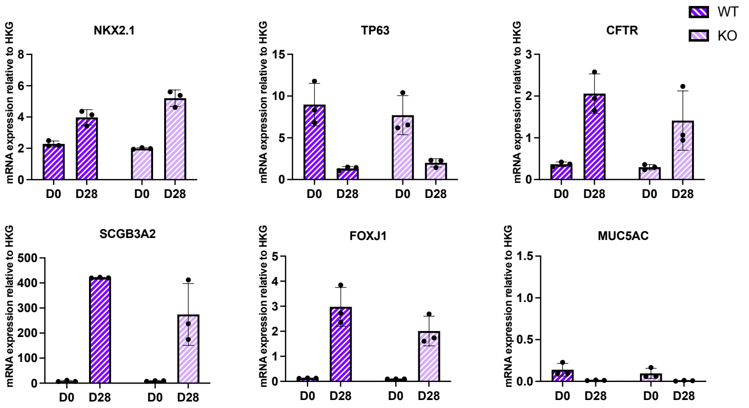

C

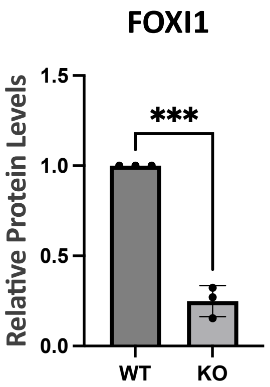

Figure S3: Additional characterisation of FOXI1 KO cells. A: FOXI1 protein sequence prediction for the FOXI1 WT and KO cell lines used in this study. Light blue highlights the DNA binding domain sequence in exon 1 whereas dark blue highlights the DNA binding domain sequence in exon 2 (transcript variant 1 only). The sequence of the epitope recognised by the anti-FOXI1 antibody used for immunofluorescence is indicated in bold. Sequences differing from WT in FOXI1 KO cells are highlighted in grey. Stop codons are represented with a red asterisk. B: Effect of 28 days of ALI differentiation on relative mRNA expression of key markers in FOXI1 WT and KO cells in genetic background 2. Filled circles represent individual values and columns are means ± SD (*n* = 3 independent experiments); differences between WT and KO are not significant, two-way ANOVA with Sidak's post-test. C: Quantification of the Western blot analysis represented in Figure 4G. Protein levels are normalised to their loading control. FOXI1 KO values are normalised to their respective WT control. Filled circles represent individual values and columns are means ± SD (*n* = 3 independent experiments), *** P<0.001; Student's t-test.

Figure S4

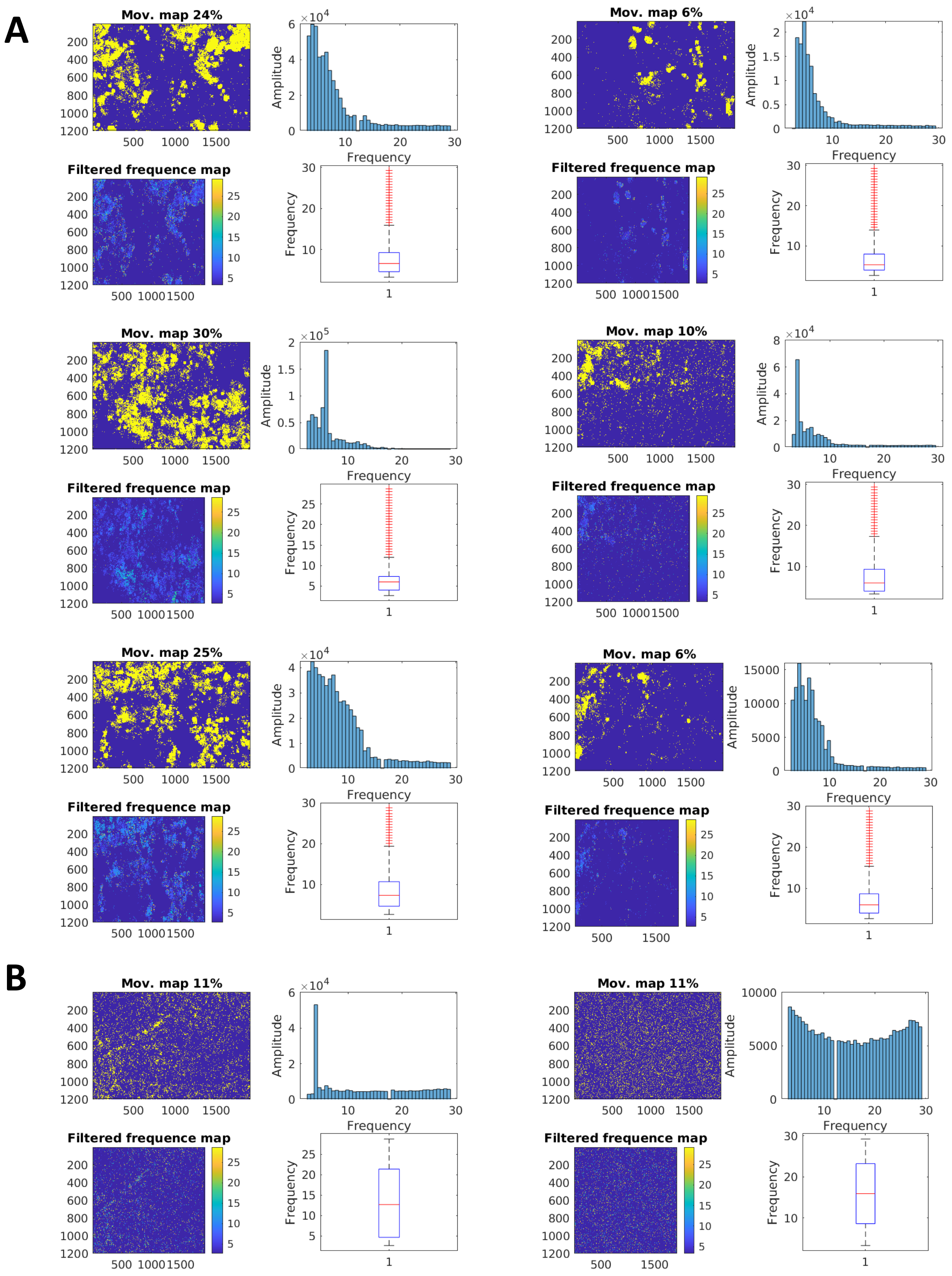

Figure S4: Representative plots of ciliary dynamics analysis. A: Representative movement and frequency plots for three fields of view (FOVs) in different samples of *FOXI1* WT ALI cultures (left) and *FOXI1* KO ALI cultures (right). B: Examples of excluded FOVs due to the presence of moving particles in the FOV (left) or background signal (right). In both cases, the percentage of cilia coverage was >2% and data points were therefore excluded.

Figure S5

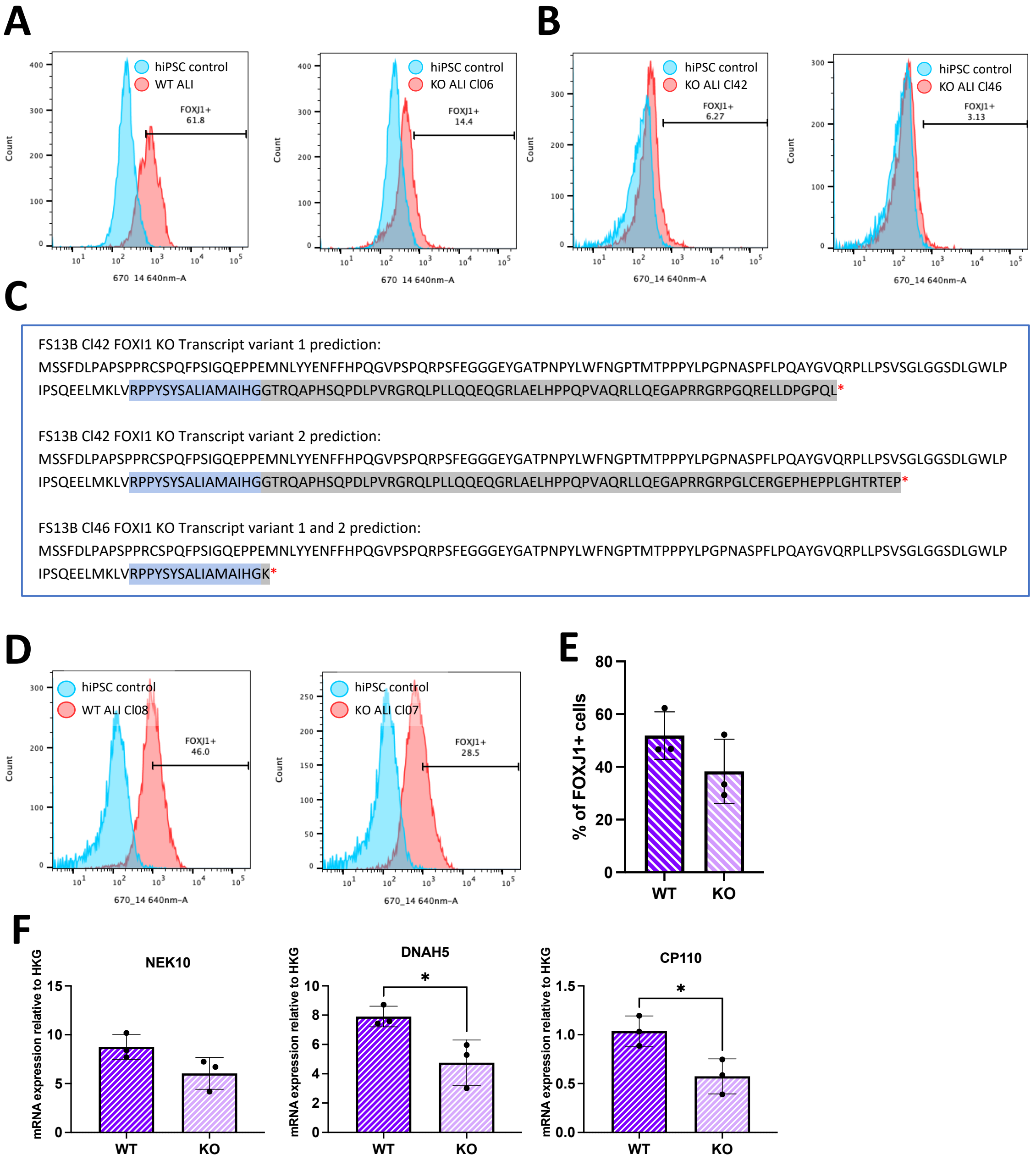

Figure S5: Flow cytometry analysis of FOXJ1 expression in hiPSC-AEC ALI cultures. A: Representative flow cytometry histograms for the analysis of FOXJ1 expression in FOXI1 WT and KO ALI cultures. B: Validation of the percentage of ciliated cells in FOXI1 KO ALI cultures in 2 additional FOXI1 KO clones from FS13B cells. C: FOXI1 protein sequence prediction for the additional FOXI1 KO clones reported in this figure. Light blue highlights DNA binding domain sequence (exon 1). Sequences differing from WT in FOXI1 KO cells are highlighted in grey. Stop codons are represented with a red asterisk. For the FOXI1 reference sequence, see Figure S3A. D: Representative flow cytometry histograms for the analysis of FOXJ1 expression in FOXI1 WT and KO ALI cultures of genetic background 2 (CF17/NKX2.1-GFP cells). The plots were generated from 9000-10000 recorded events. E: Percentage of FOXJ1 positive cells in FOXI1 WT and KO ALI cultures. Filled circles represent individual values and bars are means ± SD (*n* = 3 independent experiments); Student's t-test. F: Relative mRNA expression of mature ciliated cell markers in FOXI1 WT and KO ALI cultures. Filled circles represent individual values and bars are means ± SD (*n* = 3 independent experiments). *P < 0.05, Student's t-test.

Figure S6

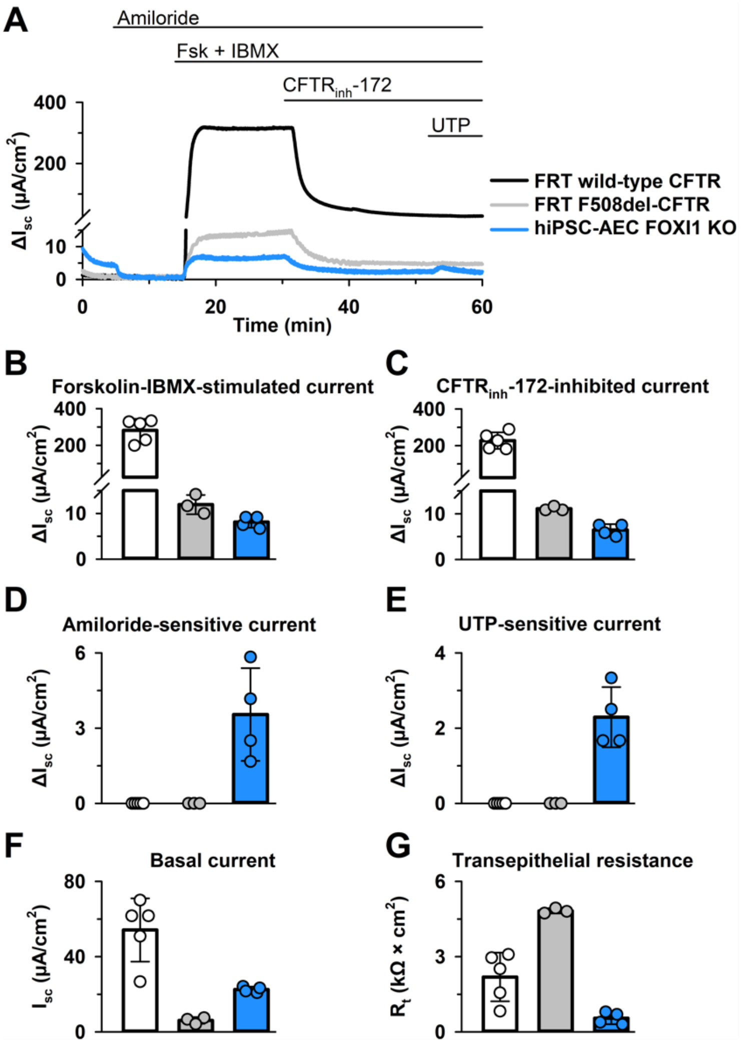

Figure S6: FOXI1 KO hiPSC-AEC epithelia functionally express CFTR, ENaC and TMEM16A. A: Representative Ussing chamber recordings from FOXI1 KO hiPSC-AECs and wild-type and F508del-CFTR-expressing FRT epithelia to show the effects of ion channel modulators on short-circuit current (I_{sc}). F508del-CFTR-expressing FRT epithelia were studied without enhancing the plasma membrane expression of F508del-CFTR by either low temperature incubation or treatment with CFTR correctors. At the indicated times, the epithelial Na^+ channel (ENaC) was inhibited with amiloride (100 μM), CFTR was activated with forskolin (Fsk; 10 μM) and 3-isobutyl-1-methylxanthine (IBMX; 100 μM) and inhibited with $CFTR_{inh}$-172 (10 μM) and the Ca^{2+}-activated Cl^- channel TMEM16A stimulated with UTP (100 μM); continuous lines indicate the presence of different compounds in the solution bathing the apical membrane of epithelia. Data are normalised to the I_{sc} value immediately preceding forskolin and IBMX addition so that ΔI_{sc} represents the change in transepithelial current after CFTR activation by Fsk and IBMX. B–E: Summary data show the magnitude of Fsk and IBMX-stimulated, $CFTR_{inh}$-172 inhibited, amiloride-sensitive and UTP-activated transepithelial currents of hiPSC-AEC and FRT epithelia expressed as ΔI_{sc}. F: Magnitude of the basal I_{sc} prior to amiloride addition for hiPSC-AEC and FRT epithelia. G: Magnitude of transepithelial resistance (R_t) measured with an epithelial voltohmmeter prior to mounting hiPSC-AEC and FRT epithelia in Ussing chambers. The R_t value of blank Millicell® Standing Cell Culture Inserts (105 ± 13 Ω cm^2; n = 2) was subtracted from those of the different epithelia studied. In B–G, symbols represent individual values and columns are means ± SD (FOXI1 KO hiPSC-AEC epithelia, n = 4; FRT-wild-type CFTR epithelia, n = 5; FRT-F508del-CFTR epithelia, n = 3). In A – C, y-axes are interrupted by a break (15–25 μA/cm^2) to magnify transepithelial currents mediated by FOXI1 KO hiPSC-AEC and FRT-F508del-CFTR epithelia.
